# Shifts in Diagnostic Testing for Headache in the Emergency Department, 2015 to 2021

**DOI:** 10.1001/jamanetworkopen.2024.7373

**Published:** 2024-04-19

**Authors:** Dustin G. Mark, Brandon H. Horton, Mary E. Reed

**Affiliations:** 1 Department of Emergency Medicine, Kaiser Permanente Medical Center, Oakland, California; 2 Department of Critical Care Medicine, Kaiser Permanente Medical Center, Oakland, California; 3 Division of Research, Kaiser Permanente Northern California, Oakland

## Abstract

**Importance:**

Subarachnoid hemorrhage is typically diagnosed by noncontrast head computed tomography (CT); lumbar puncture is recommended if computed tomography is nondiagnostic, although CT cerebral angiography has been promoted as an alternative to lumbar puncture in this diagnostic pathway. The outcomes of this debate in practice have not been studied.

**Objective:**

To determine whether CT cerebral angiography use has increased in lieu of lumbar puncture among emergency department (ED) patients with headache, with an increase in unruptured intracranial aneurysm detection.

**Design, Setting, and Participants:**

This retrospective cohort study took place in 21 community EDs of an integrated health care system in Northern California between 2015 and 2021. Participants were adult (aged >17 years) health plan members with a chief concern of headache. Exclusions were prior diagnoses of subarachnoid hemorrhage, unruptured intracranial aneurysm, cerebral arteriovenous malformation, or cerebrospinal fluid shunt. Data were analyzed from October to November 2023.

**Exposures:**

CT cerebral angiography and/or lumbar puncture during the ED encounter.

**Main Outcomes and Measures:**

Primary and secondary outcomes were 14-day and 90-day unruptured intracranial aneurysm detection, respectively. Safety outcomes were missed diagnoses of subarachnoid hemorrhage or bacterial meningitis. The annual incidence of unruptured intracranial aneurysm detection was normalized to the incidence of subarachnoid hemorrhage (UIA:SAH ratio). Average annualized percentage changes were quantified using joinpoint regression analysis.

**Results:**

Among 198 109 included ED encounters, the mean (SD) age was 47.5 (18.4) years; 140 001 patients (70.7%) were female; 29 035 (14.7%) were Black or African American, 59 896 (30.2%) were Hispanic or Latino, and 75 602 (38.2%) were White. Per year, CT cerebral angiography use increased (18.8%; 95% CI, 17.7% to 20.3%) and lumbar punctures decreased (−11.1%; 95% CI, −12.0% to −10.4%), with a corresponding increase in the 14-day UIA:SAH ratio (3.5%; 95% CI, 0.9% to 7.4%). Overall, computed tomography cerebral angiography use increased 6-fold relative to lumbar puncture, with a 33% increase in the detection of UIA. Results were similar at 90 days and robust to sensitivity analyses. Subarachnoid hemorrhage (1004 cases) and bacterial meningitis (118 cases) were misdiagnosed in 5% and 18% of cases, respectively, with no annual trends (*P* = .34; *z*_1003_ = .95 and *P* = .74; *z*_117_ = −.34, respectively).

**Conclusions and Relevance:**

In this cohort study of ED patients with headache, increases in CT cerebral angiography use were associated with fewer lumbar punctures and higher detection of unruptured intracranial aneurysms, with no significant change in missed diagnoses of subarachnoid hemorrhage or bacterial meningitis. While this shift in diagnostic strategy appeared safe in the short-term, the long-term consequences remain unclear.

## Key Points

**Question:**

Has the diagnostic evaluation of headache in the emergency department recently changed?

**Findings:**

In a cohort study of 21 emergency departments and 198 109 emergency encounters between 2015 and 2021, computed tomography cerebral angiography use increased 6-fold relative to lumbar puncture, with a 33% increase in the detection of unruptured intracranial aneurysms and no significant change in missed diagnoses of subarachnoid hemorrhage or bacterial meningitis.

**Meaning:**

These findings suggest emergency physicians are increasingly using computed tomography cerebral angiography and less often using lumbar puncture for headache evaluations, which appears safe in the short-term but has uncertain long-term consequences.

## Teaser

This cohort study assesses trends in the use of computed tomography cerebral angiography vs lumbar puncture to detect subarachnoid hemmorrhage among US emergency department patients with headache.

## Introduction

Nontraumatic subarachnoid hemorrhage (SAH) causes approximately 5% of acute-onset headaches in emergency department (ED) patients and has a 50% risk of serious disability or death at 1 year.^1,2,3,4,5^ The first-line diagnostic test for SAH is noncontrast head computed tomography (CT), which is most sensitive within the first 6 hours of headache onset.^2,6^ When CT is nondiagnostic and clinical suspicion remains high, lumbar puncture (LP) is the recommended second-line test (CT-LP approach).^7,8^ However, LP yields a high proportion of false-positive results.^9,10,11,12,13,14,15,16,17,18,19,20,21,22^ Since ruptured cerebral aneurysms account for nearly all attributable morbidity from SAH, computed tomography cerebral angiography (CTCA) has been proposed as a noninvasive alternative (CT-CTCA approach) to LP due to its relatively high sensitivity and specificity for cerebral aneurysms.^15,23,24,25,26,27,28,29^

In 2019, the American College of Emergency Physicians (ACEP) issued a weak recommendation for the use of either a CT-LP or CT-CTCA approach to exclude aneurysmal SAH.^30^ However, debate persists over the appropriateness of the CT-CTCA approach owing to concerns over cost, radiation exposure, missed alternative diagnoses (eg, meningitis), increased detection of unruptured intracranial aneurysms (UIA), and no clear advantage in cost-benefit analyses.^31,32,33,34,35,36,37,38^ Since there is little to no direct evidence supporting a CT-CTCA approach to SAH diagnosis in practice, updated 2023 guidelines from the American Heart and Stroke Associations (AHA/ASA) made a strong recommendation in favor of a CT-LP approach but no recommendation for a CT-CTCA approach.^8^

To help further inform clinical practice, we examined temporal trends in and correlations between second-line testing (LP or CTCA) for SAH and newly detected UIA among ED patients with headaches. Given rising CTCA use in the general ED patient population,^39,40^ we hypothesized that an increase in CTCA use for the evaluation of headache would be mirrored by a decrease in LP use, with an associated rise in UIA detection. From a safety perspective, we examined trends in missed diagnoses of SAH or bacterial meningitis following ED encounters for headache.

## Methods

### Study Design and Setting

We conducted a retrospective cohort study of ED encounters between January 1, 2015, and December 31, 2021, across 21 community EDs within Kaiser Permanente Northern California (KPNC). KPNC is a private, nonprofit integrated health care system with approximately 4.4 million health plan members who are representative of the regional population.^41,42^ All KPNC care settings use a comprehensive integrated electronic health record (EHR; Epic). The study was approved by the KPNC institutional review board with a waiver of informed consent because risk was minimal. We followed the Strengthening the Reporting of Observational Studies in Epidemiology (STROBE) reporting guidelines.

### Cohort Selection

We included ED encounters by adult patients (>17 years) with a chief concern of headache. We excluded encounters if there were previously established diagnoses of SAH, UIA, arteriovenous malformation of cerebral vessels, or cerebrospinal fluid shunt since these historical diagnoses may impact testing strategies or overestimate outcome incidence (eMethods in Supplement 1). We also excluded patients lacking KPNC health plan membership in the year before or the month of the ED encounter to ensure capture of comorbidities and outcomes.

### Variables

We collected the following baseline variables from the EHR: age, sex, patient-reported race and ethnicity using established categories (eMethods in Supplement 1), body mass index, initial vital signs (temperature and blood pressure), and medical history (hypertension, diabetes, hyperlipidemia, smoking, and family history of cerebral aneurysm). Race and ethnicity were evaluated in this study because of associated differential risks for SAH. The primary ED diagnosis was identified using a hierarchical list (eMethods in Supplement 1). Encounters with emergent telemedicine evaluation by a stroke neurologist (stroke alert) were identified by cross-referencing an internal registry (beginning in September 2015).^43^

### Exposures

The primary exposures of interest were CTCA or LP during the ED encounter. We also determined whether CT was done in the ED as well as whether digital subtraction cerebral angiography (DSA) was performed within 72 hours following an ED encounter. A diagnostic testing subcohort, defined as encounters with either CT, CTCA, or LP, was created to enrich the dataset for nonbenign presentations. Exposures were determined based on internal procedural codes, laboratory tests (LP), and procedural notes (DSA).

### Outcomes

The primary outcome was detection of a UIA within 14 days of an index ED encounter, defined as either a new UIA diagnosis on the problem list or notation of a cerebral aneurysm within the text of a radiology report, as determined by natural language processing (positive predictive value of 98.7%) (eMethods in Supplement 1). Secondary outcomes were UIA detection within 90 days and SAH within 14 and 90 days. SAH was defined as a clinical encounter (including external claims data) with a coded diagnosis of nontraumatic SAH. Of note, UIA and SAH outcomes were mutually exclusive; patients with a diagnosis of SAH were excluded from UIA counts both to avoid inclusion of ruptured aneurysms detected by natural language processing and because UIA discovered coincident to ruptured aneurysms have different clinical implications.^44^ We chose a 14-day window for the primary outcome both to account for differences in follow-up studies associated with ED diagnostics and to allow for normalization of UIA prevalence using SAH incidence, since most delayed diagnoses of SAH are made within 2 weeks of initial presentation and risk factors for UIA and SAH overlap.^4,38,45,46^

To assess the safety of shifts in diagnostic strategies, we manually reviewed medical records with potential missed diagnoses of SAH or bacterial meningitis and documented the diagnostic strategy used at the index ED encounter (no testing, CT only, CTCA, or LP). A potential missed diagnosis of SAH was assigned if SAH was noted within 14 days following an index ED encounter with neither a linked inpatient hospitalization nor an ED diagnosis of SAH.^47^ A potential missed diagnosis of bacterial meningitis was similarly defined, except a 7-day window was used.

### Statistical Analysis

Annualized rates of test utilization (CT, CTCA, LP, and DSA) were calculated on a per-encounter basis, including annualized ratios of CTCA to LP rates (CTCA:LP ratio). Outcomes were calculated on a per-person basis (indexed to the year of the ED visit) and summarized as an annualized ratio of UIA to SAH incidence (UIA:SAH ratio). Primary analyses were performed using both the full study cohort and the diagnostic testing subcohort.

We examined trends in test utilization and outcomes with joinpoint regression analysis using cluster-robust variance estimators to account for within-ED correlations. Joinpoints were determined by fitting least squares regression lines to the natural logarithm of the rates with calendar year as a regressor variable, accounting for trend transitions, with a maximum of 1 joinpoint. Trends were reported as the average annualized percentage change (AAPC). The AAPC was calculated using the weighted average of slope coefficients and statistical significance was reported using 95% CIs, where exclusion of 0 from the CI indicates rejection of the null hypothesis.^48^ All other trends were assessed using the Cuziak nonparametric test for trend across ordered groups reported as *P* values using a 2-sided test with results deemed statistically significant at *P* values less than .05.^49^ We used the Pearson product-moment correlation coefficient to assess the degree of correlation between annualized CTCA:LP and UIA:SAH ratios. We defined absolute correlation coefficients between 0.70 to 0.89 as indicators of a strong correlation and 0.90 or greater as indicators of a very strong correlation.^50^ Data analyses were performed using Stata version 14.1 (StataCorp) and Joinpoint version 5.0 (National Cancer Institute). Data were analyzed from October to November 2023.

#### Sensitivity Analysis

We repeated all analyses after excluding encounters from 2015 or with ED stroke alerts since diagnoses using *International Classification of Diseases, Ninth Revision* codes (in use through September 2015) may have changed outcome counts, and stroke alerts, which began in September 2015, may have been associated with increases in CTCA use.^43^ We also assessed for bias due to between-ED differences using Poisson regression with dummy variables for each ED and robust variance estimators. Finally, we examined alternative UIA outcomes by (1) only including problem list entries of UIA and (2) calculating the risk-adjusted marginal incidence of UIA using Poisson regression as above with additional covariates for age, sex, race and ethnicity, diabetes, hypertension, smoking status, and family history of cerebral aneurysms, all selected per clinical rationale.^45,51,52^

## Results

**Figure 1.**
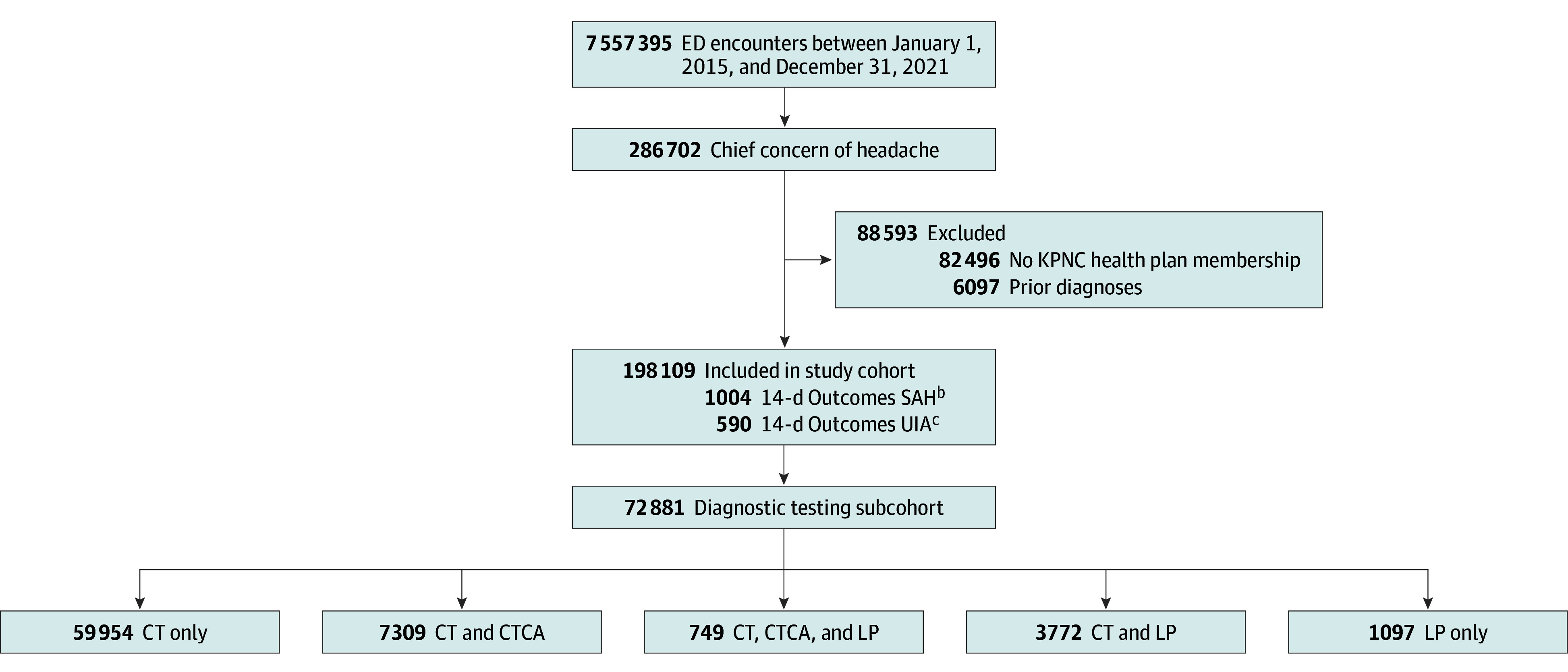
Study Cohort Assembly CT indicates noncontrast head computed tomography; CTCA, computed tomography cerebral angiography; ED, emergency department; KPNC, Kaiser Permanente Northern California; LP, lumbar puncture; SAH, subarachnoid hemorrhage; UIA, unruptured intracranial aneurysm. ^a^History of unruptured cerebral aneurysm (n = 2209), ventricular shunt (n = 1083), subarachnoid hemorrhage (n = 1368), cerebral arteriovenous malformation (n = 421), multiple exclusions (n = 1016). ^b^International Classification of Diseases 9th or 10th edition diagnosis for SAH within 14 days following eligible ED encounter. ^c^Entry of UIA on problem list or angiography report with a suspected cerebral aneurysm within 14 days of an eligible ED encounter, exclusive of contemporaneous SAH diagnoses.

A total of 198 109 encounters were included in the full study cohort. The mean (SD) age was 47.5 (18.4) years; 140 001 patients (70.7%) were female; 29 035 (14.7%) were Black or African American, 59 896 (30.2%) were Hispanic or Latino, and 75 602 (38.2%) were White; 74 436 (37.6%) had hypertension; and headache, SAH, and meningitis accounted for 116 807 (58.9%), 901 (0.5%), and 699 (0.4%) of ED diagnoses, respectively. There were 72 881 (36.8%) encounters in the diagnostic testing subcohort. Full demographics, baseline characteristics, and ED diagnoses are shown in Table 1, and are shown by year in eTable 1 in Supplement 1. Overall there were 7 557 395 ED encounters during the study period, of which 286 702 (3.8%) had a chief concern of headache (Figure 1).

**Table 1.** Demographics, Baseline Characteristics, and Diagnoses During Emergency Department (ED) Encounters for Headache, 2015 to 2021

Characteristic	Encounters, No. (%)	
	Full study cohort (n = 198 109)	Diagnostic testing subcohort (n = 72 881)^a^
Age, mean (SD), y	47.5 (18.4)	54.3 (18.9)
Sex		
Female	140 001 (70.7)	47 950 (65.8)
Male	58 108 (29.3)	24 931 (34.2)
Race		
Asian	26 242 (13.2)	10 603 (14.5)
Black/African American	29 035 (14.7)	9001 (12.4)
Hispanic/Latino	59 896 (30.2)	19 958 (27.4)
White	75 602 (38.2)	30 813 (42.3)
Other^b^	7334 (3.7)	2506 (3.4)
Body mass index, mean (SD)^c^	29.0 (6.0)	28.8 (5.8)
Hypertension	74 436 (37.6)	35 030 (48.1)
Smoking		
Active	8197 (4.1)	2840 (3.9)
Former	41 348 (20.9)	17 850 (24.5)
Never	129 563 (65.4)	46 107 (63.3)
Unknown	19 001 (9.6)	6084 (8.3)
Diabetes	35 894 (18.1)	16 960 (23.3)
Hyperlipidemia	67 665 (34.2)	32 360 (44.8)
Family history of cerebral aneurysm	186 (0.1)	88 (0.1)
Temperature >100.4 °F	1681 (0.8)	234 (0.3)
Systolic blood pressure, mean (SD), mmHg	130.9 (19.9)	133.6 (20.5)
Diastolic blood pressure, mean (SD), mmHg	75.0 (13.2)	74.4 (13.2)
Stroke alert activation in ED	1030 (0.5)	992 (1.4)
Admission to hospital	13 674 (6.9)	8477 (11.6)
ED diagnosis		
Headache	116 807 (58.9)	54 625 (75.0)
Migrane	22 347 (11.3)	1822 (2.5)
Subarachnoid hemorrhage	901 (0.5)	867 (1.2)
Hypertension	10 294 (5.2)	2125 (2.9)
Upper respiratory infection	5199 (2.6)	231 (0.3)
Brain tumor	703 (0.4)	476 (0.7)
Intracerebral hemorrhage	374 (0.2)	346 (0.5)
Meningitis	699 (0.4)	634 (0.9)
Ischemic stroke	3489 (1.8)	3095 (4.2)
Other	37 296 (18.8)	8660 (11.9)

^a^ The diagnostic testing subcohort was restricted to encounters during which there was testing with either noncontrast head computed tomography, computed tomography cerebral angiography or lumbar puncture.

^b^ Includes American Indian/Alaska Native, Native Hawaiian/Other Pacific Islander, declined to state, unknown, and other.

^c^ Body mass index is calculated as weight in kilograms divided by height in meters squared.

**Figure 2.**
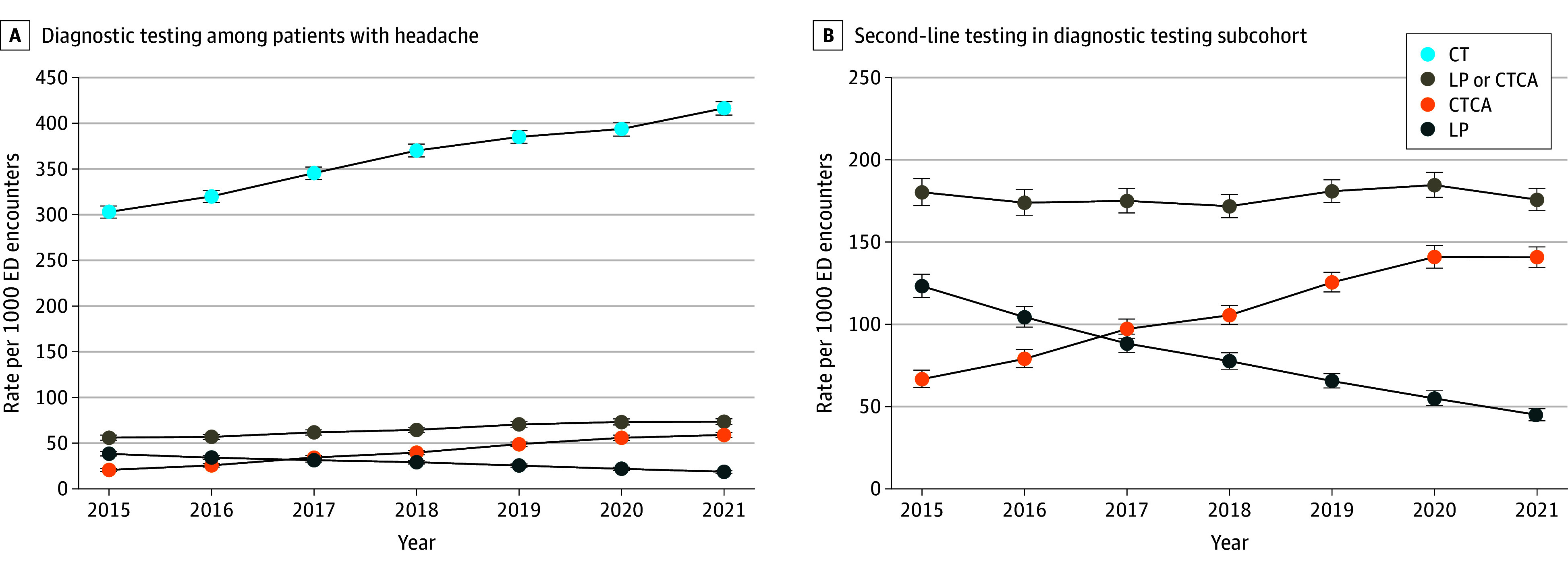
Trends in Diagnostic Testing Vertical bars represent 95% CIs. CT indicates computed tomography; CTCA, computed tomography cerebral angiography; ED, emergency department; LP, lumbar puncture.

There was a year-to-year increase in CT use, with an AAPC of 5.4% (95% CI, 5.1% to 5.8%) (Table 2). At the same time, there were diverging trends in second-line testing (LP and CTCA) (Figure 2) with a year-to-year increase in CTCA (AAPC, 18.8%; 95% CI, 17.7% to 20.3%) and a corresponding decrease in LP (AAPC, −11.1%; 95% CI, −12.0% to −10.4%). These shifts in second-line test choice resulted in a marked increase in the CTCA:LP ratio (AAPC, 35.0%; 95% CI, 32.6% to 37.2%) as well as increases in overall second-line testing (AAPC, 5.3%; 95% CI, 3.7% to 7.0%). Diagnostic testing subcohort analysis revealed smaller increases in CTCA use and larger decreases in LP, with no significant change in overall second-line testing (AAPC, 0.4%; 95% CI, −1.5% to 2.4%). In neither analysis was there a significant change in 72-hour DSA use. Sensitivity analyses excluding 2015 encounters and stroke alerts yielded consistent results (eTable 2 in Supplement 1), as did analysis using Poisson regression, with the exception that a decrease in 72-hour DSA use became statistically significant in the diagnostic testing subcohort (APC, −4.9%; 95% CI, −9.2% to −0.5%) (eTable 3 in Supplement 1). Analysis by age strata revealed that increases in CT utilization were progressively greater for younger patients (AAPC, 6.9% for age 18-40 vs 2.8% for age >80 years) (eTable 4 and eFigure 1 in Supplement 1), whereas CTCA increases were relatively smaller in those aged 61 to 80 years (eTable 5 and eFigure 2 in Supplement 1). Declines in LP were only observed among encounters with patients aged 80 years and younger (eTable 6 and eFigure 3 in Supplement 1).

**Table 2.** Trends in Test Utilization During Emergency Department Encounters for Headache, 2015 to 2021

Test	Utilization rate (per 1000 encounters)							AAPC (95% CI)
	2015	2016	2017	2018	2019	2020	2021	
Full study cohort								
Encounters, No.	27 132	27 944	28 489	29 179	31 090	24 942	29 333	NA
CT	302.8	319.9	345.3	370.0	385.0	393.5	416.3	5.4 (5.1 to 5.8)^a^
CTCA	20.7	25.8	34.3	39.7	48.9	55.9	58.9	18.8 (17.7 to 20.3)^a^
LP	38.3	34.1	31.1	29.2	25.5	21.8	18.8	−11.1 (−12.0 to −10.4)^a^
Either LP or CTCA	56.1	56.8	61.7	64.5	70.5	73.3	73.6	5.3 (3.7 to 7.0)^b^
Both LP and CTCA	3.0	3.1	3.7	4.3	3.9	4.4	4.1	6.4 (3.6 to 10.1)^a^
CTCA:LP ratio	0.5	0.8	1.1	1.4	1.9	2.6	3.1	35.0 (32.6 to 37.2)^a^
DSA within 72 h	2.7	3.3	3.2	1.8	2.2	3.2	2.7	−2.4 (−13.7 to 8.9)^b^
Diagnostic testing subcohort								
Encounters, No.	8442	9132	10 045	10 969	12 115	9895	12 283	NA
CT	973.1	979.0	979.2	984.3	988.1	991.8	994.1	0.3 (0.3 to 0.4)^b^
CTCA	66.6	79.0	97.2	105.5	125.5	140.9	140.7	13.2 (11.7 to 15.8)^a^
LP	123.2	104.4	88.3	77.6	65.5	54.9	44.9	−15.3 (−16.5 to −14.3)^a^
Either LP or CTCA	180.2	173.9	175.0	171.7	180.9	184.6	175.7	0.4 (−1.5 to 2.4)^b^
Both LP and CTCA	9.6	9.4	10.5	11.4	10.0	11.1	9.9	1.2 (−1.4 to 4.9)^a^
CTCA:LP ratio	0.5	0.8	1.1	1.4	1.9	2.6	3.1	35.0 (32.6 to 37.2)^a^
DSA within 72 h	7.7	9.5	8.4	4.7	5.2	7.8	6.3	−6.1 (−17.9 to 6.2)^b^

Abbreviations: AAPC, average annual percentage change; CT, noncontrast head computed tomography; CTCA, computed tomography cerebral angiography; DSA, digital subtraction invasive cerebral angiography; LP, lumbar puncture; NA, not applicable.

^a^ One joinpoint used in average calculation.

^b^ No joinpoints used in average calculation (see eTable 11 in Supplement 1).

Outcomes are summarized in Table 3. Most outcomes occurred within 3 days of an index encounter (eFigure 4 and eFigure 5 in Supplement 1). For the primary outcome of 14-day UIA detection, there was an increase in the 14-day UIA:SAH ratio in both the full study cohort (AAPC, 3.5%; 95% CI, 0.9% to 7.4%) and the diagnostic testing subcohort (AAPC, 7.8%; 95% CI, 4.2% to 16.4%). Similar trends were seen for the 90-day UIA:SAH ratio, and sensitivity analyses were consistent with primary analyses (eTable 7, eTable 8, eTable 9, and eTable 10 in Supplement 1). Joinpoint segment APCs for the primary analyses are shown in eTable 11 and eTable 12 in Supplement 1.

**Table 3.** Primary and Secondary Outcomes Among Emergency Department Patients With Headache, 2015 to 2021

Year	Incidence rate (per 1000 person)							AAPC (95% CI)
	2015	2016	2017	2018	2019	2020	2021	
Full study cohort								
Persons, No.	23 010	24 215	25 118	25 785	27 554	22 484	26 312	NA
14-d incidence								
UIA	3.0	2.9	2.7	3.5	3.0	4.4	4.1	7.5 (1.2 to 16.2)^a^
SAH	4.7	5.8	5.9	6.2	5.5	6.8	5.4	2.4 (−5.6 to 12.0)^a^
UIA:SAH ratio	0.6	0.5	0.5	0.6	0.6	0.6	0.8	3.5 (0.9 to 7.4)^b^
90-d incidence								
UIA	3.5	3.8	3.4	4.0	3.9	5.1	4.8	6.6 (1.2 to 13.6)^a^
SAH	5.1	6.2	6.3	6.6	6.0	7.1	5.7	2.0 (−5.0 to 10.6)^a^
UIA:SAH ratio	0.7	0.6	0.5	0.6	0.7	0.7	0.8	3.0 (2.8 to 3.9)^b^
Diagnostic test subcohort								
Persons, No.	7660	8333	9216	10 066	11 069	9111	11 311	NA
14-d incidence								
UIA	6.5	6.1	6.4	7.6	6.8	9.4	9.0	7.4 (2.2 to 14.9)^a^
SAH	13.3	15.5	15.6	14.7	12.8	15.6	12.2	−1.3 (−10.2 to 10.4)^a^
UIA:SAH ratio	0.5	0.4	0.4	0.5	0.5	0.6	0.7	7.8 (4.2 to 16.4)^b^
90-d incidence								
UIA	6.9	7.6	7.7	8.3	8.1	10.8	10.1	7.1 (4.2 to 10.9)^a^
SAH	13.8	16.6	16.6	15.5	13.9	16.1	12.8	−1.6 (−9.4 to 9.1)^a^
UIA:SAH ratio	0.5	0.5	0.5	0.5	0.6	0.7	0.8	8.0 (6.3 to 12.1)^b^

Abbreviations: AAPC, average annual percentage change; NA, not applicable; SAH, nontraumatic subarachnoid hemorrhage; UIA, unruptured intracranial aneurysm.

^a^ No joinpoints used in average calculation (see eTable 12 in Supplement 1).

^b^ One joinpoint used in average calculation.

Annualized CTCA:LP ratios and UIA:SAH ratios were strongly correlated in the full study cohort (*r* = 0.70) (eFigure 6 in Supplement 1) and very strongly correlated in the diagnostic testing subcohort (*r* = 0.90) (eFigure 7 in Supplement 1). Sensitivity analyses further strengthened these correlations (*r* = 0.93 for the full study cohort and 0.96 for the diagnostic testing subcohort) (eFigure 8 and eFigure 9 in Supplement 1). Post hoc analysis of outcomes occurring within 1 day of an index ED encounter (eTable 13 in Supplement 1) showed steady increases in evaluations that included CTCA, both for SAH (range, 45.8% to 73.6%; *P* < .001; *z*_900_ = 4.69) and UIA (range, 44.2% to 82.4%; *P* < .001; *z*_496_ = 7.18).

There were 50 possible missed diagnoses of SAH, comprising 5.0% (95% CI, 3.8% to 6.5%) of 14-day SAH cases with no annual trend (*P* = .34; *z*_1003_ = .95) (eTable 14 in Supplement 1). Testing for SAH during the index ED encounter was none in 14 encounters (28.0%), CT only in 29 (58.0%), CTCA in 6 (12.0%), and LP in 1 (2.0%). Of the 7 encounters with second-line testing (eTable 15 in Supplement 1), 3 had a subsequent aneurysmal SAH within 14 days: in 1 case, CT and CTCA were both interpreted as normal, and in 2 cases, CT was negative for SAH and CTCA was positive for aneurysm, but both patients declined further recommended evaluation with LP. There were 21 possible missed diagnoses of bacterial meningitis, comprising 17.8% (95% CI, 12.0% to 25.7%) of 7-day bacterial meningitis cases with no annual trend (*P* = .73; *z*_117_ = −.34) (eTable 16 in Supplement 1). Testing during the index ED encounter was none in 10 encounters (48%), CT only in 9 (43%), CTCA in 0 (0%) and LP in 1 (5%). The 1 case with second-line testing had culture-confirmed pneumococcal meningitis with normal initial findings on cerebrospinal fluid analysis (eTable 17 in Supplement 1).

## Discussion

This cohort study is novel in quantifying a recent shift away from CT-LP and toward CT-CTCA approaches to ED patients with headache. Additionally, to our knowledge, this is the first study to assess changes in UIA detection with increasing CTCA use. The discussion will explore plausible reasons for and potential implications of this shift in diagnostic strategy.

We observed an 18.8% annualized increase in CTCA use; by 2021, CTCA was used in 5.9% of ED headache encounters, comparable with other US ED settings.^53^ While some of this rise was due to increasing CTCA use in ischemic stroke evaluations, this appeared to be a minor factor since changes in CTCA rates were greater than ED diagnoses of ischemic stroke. Rather, a corresponding 11.1% average annual decrease in LP, alongside unchanged overall second-line testing rates in the diagnostic testing subcohort, suggests that clinicians were largely substituting one test for another, even if some of the decrease in LP was due to incorporation of CT-only approaches to SAH diagnosis.^54^ This is also consistent with cross-sectional surveys of emergency physicians regarding SAH diagnostic strategies, which suggest rising acceptance of CTCA over LP, with a majority preference for CTCA in US settings.^55,56,57^

The 2019 ACEP endorsement of CTCA as a substitute for LP was based on very high (97%-98%) overall sensitivity and specificity of CTCA for cerebral aneurysms, including case-series suggesting 100% sensitivity of CTCA for causative aneurysms in patients with CT-negative/LP-positive SAH.^10,23,30,35,36,37,58,59,60^ One criticism of a CT-CTCA approach is lower sensitivity for very small aneurysms (≤3 mm), which account for up to 18% of ruptured aneurysms, whereas CT-LP is essentially 100% sensitive for aneurysmal SAH.^11,12,25,26,27,61,62,63^ These differences in test characteristics, however, must be balanced against the low incidence of CT-negative aneurysmal SAH in clinical practice.^9,13,14,20,21^ Taking a Bayesian approach and a conservative 1% probability of CT-negative aneurysmal SAH, a test with 90% sensitivity and specificity yields a negative likelihood ratio of 0.11 and a negative predictive value (NPV) of 99.89% (ie, approximately 1 in 900 risk). In comparison, assuming a 10% probability of SAH and a headache onset within 6 hours, for which CT has 98.7% sensitivity and 100% specificity for SAH, the likelihood ratio of a negative CT is 0.013, yielding an NPV of 99.86% (approximately 1 in 700 risk).^8,12^ As such, with comparable estimated false negative rates between CT-CTCA and (guideline-endorsed) 6-hour CT approaches, and considering a 6% risk of significant LP-related complications, clinician preference for a CT-CTCA approach is understandable.^8,13,56^

There are likely circumstances in which the probability of CT-negative SAH is high enough that LP is justified, such as delayed presentations of thunderclap headache, and differential diagnoses must be considered since SAH represents a minority (<15%) of pathologies detected by LP.^1,12,21,64,65,66,67^ However, while a CT-LP approach was given a class 1 recommendation by the AHA/ASA, they also did not explicitly recommended against a CT-CTCA approach. Accordingly, despite the low level of evidence behind the ACEP recommendation, it would seem that clinicians can safely offer a CT-CTCA approach to selected patients, a conclusion reinforced by the lack of increases in missed diagnoses of SAH or bacterial meningitis in our study setting.

Ultimately the principal consequence of selecting a CT-CTCA approach is increased UIA detection, as we observed. The natural history of UIAs discovered during symptomatic workups is not well understood, with future risk estimation confounded by patient selection for preemptive treatment.^68^ Given this uncertainty, concerns have been raised over the psychological burden of UIA detection.^35^ Ultimately, whether detection of UIA is a burden or a benefit remains to be determined but is likely similar to other aneurysmal arterial diseases (eg, aortic) in that the absolute risk of rupture is low, the consequences of rupture are severe, and preemptive intervention is potentially harmful.

Other notable findings include a 5.4% average annual increase in CT use, which also appeared to be associated with an increase in overall second-line testing. Finally, while not reproducible across analyses, the significant decrease in 72-hour DSA utilization seen in the diagnostic testing subcohort with Poisson regression sensitivity analysis could be attributed to increasing use of CTCA to exclude aneurysmal causes of CT-negative and perimesencephalic SAH.^9,69^ Ultimately, the small absolute number of DSAs performed limited the detection of significant changes.

### Limitations

This study has limitations. As a retrospective study using diagnostic codes to define outcomes, the results are subject to coding errors and incomplete outcome capture. However, assuming that misclassifications were equally distributed over time, the temporal basis of analysis mitigated these risks.^1,37,66,70^ Although we did not assess magnetic resonance imaging, since this is not a recommended screening modality for SAH and had variable availability between EDs and over time, a proportion of UIAs were ostensibly diagnosed using magnetic resonance angiography. However, it is likely that this impact on UIA detection decreased over time as the proportion of UIA outcomes involving CTCA in the ED steadily increased.

We also observed that 5% of SAH and 18% of bacterial meningitis cases were possibly misdiagnosed at index ED visits, estimates which are consistent with prior literature.^3,47,71^ Although we did not observe a significant change in the proportion of missed diagnoses over time, the small number of cases limited detection of differences. It is also notable that ED diagnoses of meningitis decreased slightly over time. Whether this was due to shifting epidemiologic trends and/or undiagnosed nonbacterial meningitis is unclear, though most meningitis diagnoses are attributable to viral causes for which delays in treatment do not clearly effect outcomes.^21,67,72^

## Conclusions

In this retrospective study of diagnostic evaluations for ED patients with headache within 21 community EDs of an integrated health care system between 2015 and 2021, we observed a steady increase in CTCA rates and a corresponding decrease in LP rates, with a small but statistically significant increase in the detection of UIA. This shift in testing strategies appeared safe in that it did not appreciably raise the risk of missed diagnoses of SAH or bacterial meningitis. However, the longer-term impact and costs of this shift in diagnostic strategy remain unclear.

## Notes

Supplement 1.
eMethods.
**eTable 1. **Demographics, Baseline Characteristics, and Diagnoses: Emergency Department Encounters for Headache, 2015 to 2021, Full Study Cohort **eTable 2. **Sensitivity Analysis: Trends in Test Utilization During Emergency Department Encounters for Headache, Excluding Stroke Alert Activations and 2015 Encounters **eTable 3. **Sensitivity Analysis: Comparison of Trends in Test Utilization During Emergency Department Encounters for Headache Using Joinpoint Analysis vs Poisson Regression Adjusted for Emergency Department-Level Effects **eTable 4. **Time Trends in Noncontrast Computed Tomography (CT) of the Head During Emergency Department Encounters for Headache (2015-2021), Stratified by Age **eFigure 1.** Time Trends in Noncontrast Computed Tomography (CT) of the Head, Stratified by Age **eTable 5. **Time Trends in Computed Tomography Cerebral Angiography (CTCA) During Emergency Department Encounters for Headache (2015-2021), Stratified by Age **eFigure 2. **Time Trends in Computed Tomography Cerebral Angiography (CTCA), Stratified by Age **eTable 6. **Time Trends in Lumbar Puncture (LP) During Emergency Department Encounters for Headache (2015-2021), Stratified by Age **eFigure 3. **Time Trends in Lumbar Puncture (LP), Stratified by Age **eFigure 4. **Days From Index ED Encounter to Subarachnoid Hemorrhage (SAH) Diagnosis **eFigure 5. **Days From Index ED Encounter to Unruptured Intracranial Aneurysm (UIA) Diagnoses **eTable 7. **Sensitivity Analysis: Primary and Secondary Outcomes, Excluding Stroke Alert Activations and 2015 Encounters **eTable 8. **Sensitivity Analysis: Unruptured Intracranial Aneurysm (UIA) Detection by Problem List Only **eTable 9. **Sensitivity Analysis: Risk-Adjusted Unruptured Intracranial Aneurysm (UIA) Rates Among Emergency Department Patients With Headache, 2015 to 2021 **eTable 10. **Sensitivity Analysis: Comparison of Trends in Test Utilization During Emergency Department Encounters for Headache Using Joinpoint Analysis Versus Poisson Regression Adjusted for Emergency Department-Level Effects **eTable 11. **Average Percentage Change (APC) in Test Utilization by Joinpoint Segment, Primary Analysis **eTable 12. **Average Percentage Change (APC) in Outcomes by Joinpoint Segment, Primary Analysis **eFigure 6. **Correlation Between Annualized CTCA:LP Ratios and 14-Day UIA:SAH Ratios (Full Study Cohort) **eFigure 7. **Correlation Between Annualized CTCA:LP Ratios and 14-Day UIA:SAH Ratios (Diagnostic Testing Subcohort) **eFigure 8.** Correlation Between Annualized CTCA:LP Ratios and 14-Day UIA:SAH Ratios (Full Study Cohort, Sensitivity Analysis Excluding Stroke Alert Activations and 2015 Encounters) **eFigure 9. **Correlation Between Annualized CTCA:LP Ratios and 14-Day UIA:SAH Ratios (Diagnostic Testing Subcohort, Sensitivity Analysis Excluding Stroke Alert Activations and 2015 Encounters) **eTable 13. **Diagnostic Testing During ED Encounters With Study Outcomes Within 1 Calendar Day **eTable 14. **Possible Missed Diagnoses of Subarachnoid Hemorrhage (SAH) **eTable 15. **Manual Medical Record Review of Possible Missed Diagnoses of SAH With Secondary Testing **eTable 16. **Possible Missed Diagnoses of Bacterial Meningitis **eTable 17. **Manual Medical Record Review of Possible Missed Diagnoses of Bacterial Meningitis Following Secondary Testing

Supplement 2.
Data Sharing Statement
