# Supplementary material for: Shifts in Diagnostic Testing for Headache in the Emergency Department, 2015 to 2021
Source: JAMA Netw Open. 2024 Apr 19;7(4):e247373. doi: 10.1001/jamanetworkopen.2024.7373 (PMC11031686; doi:10.1001/jamanetworkopen.2024.7373)
Supplement: Supplement 1. — eMethods. eTable 1. Demographics, Baseline Characteristics, and Diagnoses: Emergency Department Encounters for Headache, 2015 to 2021, Full Study Cohort eTable 2. Sensitivity Analysis: Trends in Test Utilization During Emergency Department Encounters for Headache, Excluding Stroke Alert Activations and 2015 Encounters eTable 3. Sensitivity Analysis: Comparison of Trends in Test Utilization During Emergency Department Encounters for Headache Using Joinpoint Analysis vs Poisson Regression Adjusted for Emergency Department-Level Effects eTable 4. Time Trends in Noncontrast Computed Tomography (CT) of the Head During Emergency Department Encounters for Headache (2015-2021), Stratified by Age eFigure 1. Time Trends in Noncontrast Computed Tomography (CT) of the Head, Stratified by Age eTable 5. Time Trends in Computed Tomography Cerebral Angiography (CTCA) During Emergency Department Encounters for Headache (2015-2021), Stratified by Age eFigure 2. Time Trends in Computed Tomography Cerebral Angiography (CTCA), Stratified by Age eTable 6. Time Trends in Lumbar Puncture (LP) During Emergency Department Encounters for Headache (2015-2021), Stratified by Age eFigure 3. Time Trends in Lumbar Puncture (LP), Stratified by Age eFigure 4. Days From Index ED Encounter to Subarachnoid Hemorrhage (SAH) Diagnosis eFigure 5. Days From Index ED Encounter to Unruptured Intracranial Aneurysm (UIA) Diagnoses eTable 7. Sensitivity Analysis: Primary and Secondary Outcomes, Excluding Stroke Alert Activations and 2015 Encounters eTable 8. Sensitivity Analysis: Unruptured Intracranial Aneurysm (UIA) Detection by Problem List Only eTable 9. Sensitivity Analysis: Risk-Adjusted Unruptured Intracranial Aneurysm (UIA) Rates Among Emergency Department Patients With Headache, 2015 to 2021 eTable 10. Sensitivity Analysis: Comparison of Trends in Test Utilization During Emergency Department Encounters for Headache Using Joinpoint Analysis Versus Poisson Regression Adjusted for Emergency Department-Lev [file jamanetwopen-e247373-s001.pdf]

Supplemental Online Content

Mark DG, Horton BH, Reed ME. Shifts in diagnostic testing for headache in the emergency department, 2015 to 2021. *JAMA Netw Open*. 2024;7(4):e247373. doi:10.1001/jamanetworkopen.2024.7373

eMethods

eTable 1. Demographics, Baseline Characteristics, and Diagnoses: Emergency Department Encounters for Headache, 2015 to 2021, Full Study Cohort

eTable 2. Sensitivity Analysis: Trends in Test Utilization During Emergency Department Encounters for Headache, Excluding Stroke Alert Activations and 2015 Encounters

eTable 3. Sensitivity Analysis: Comparison of Trends in Test Utilization During Emergency Department Encounters for Headache Using Joinpoint Analysis vs Poisson Regression Adjusted for Emergency Department-Level Effects

eTable 4. Time Trends in Noncontrast Computed Tomography (CT) of the Head During Emergency Department Encounters for Headache (2015-2021), Stratified by Age

eFigure 1. Time Trends in Noncontrast Computed Tomography (CT) of the Head, Stratified by Age

eTable 5. Time Trends in Computed Tomography Cerebral Angiography (CTCA) During Emergency Department Encounters for Headache (2015-2021), Stratified by Age

eFigure 2. Time Trends in Computed Tomography Cerebral Angiography (CTCA), Stratified by Age

eTable 6. Time Trends in Lumbar Puncture (LP) During Emergency Department Encounters for Headache (2015-2021), Stratified by Age

eFigure 3. Time Trends in Lumbar Puncture (LP), Stratified by Age

eFigure 4. Days From Index ED Encounter to Subarachnoid Hemorrhage (SAH) Diagnosis

eFigure 5. Days From Index ED Encounter to Unruptured Intracranial Aneurysm (UIA) Diagnoses

eTable 7. Sensitivity Analysis: Primary and Secondary Outcomes, Excluding Stroke Alert Activations and 2015 Encounters

eTable 8. Sensitivity Analysis: Unruptured Intracranial Aneurysm (UIA) Detection by Problem List Only

© 2024 Mark DG et al. *JAMA Network Open.*

eTable 9. Sensitivity Analysis: Risk-Adjusted Unruptured Intracranial Aneurysm (UIA) Rates Among Emergency Department Patients With Headache, 2015 to 2021

eTable 10. Sensitivity Analysis: Comparison of Trends in Test Utilization During Emergency Department Encounters for Headache Using Joinpoint Analysis Versus Poisson Regression Adjusted for Emergency Department-Level Effects

eTable 11. Average Percentage Change (APC) in Test Utilization by Joinpoint Segment, Primary Analysis

eTable 12. Average Percentage Change (APC) in Outcomes by Joinpoint Segment, Primary Analysis

eFigure 6. Correlation Between Annualized CTCA:LP Ratios and 14-Day UIA:SAH Ratios (Full Study Cohort)

eFigure 7. Correlation Between Annualized CTCA:LP Ratios and 14-Day UIA:SAH Ratios (Diagnostic Testing Subcohort)

eFigure 8. Correlation Between Annualized CTCA:LP Ratios and 14-Day UIA:SAH Ratios (Full Study Cohort, Sensitivity Analysis Excluding Stroke Alert Activations and 2015 Encounters)

eFigure 9. Correlation Between Annualized CTCA:LP Ratios and 14-Day UIA:SAH Ratios (Diagnostic Testing Subcohort, Sensitivity Analysis Excluding Stroke Alert Activations and 2015 Encounters)

eTable 13. Diagnostic Testing During ED Encounters With Study Outcomes Within 1 Calendar Day

eTable 14. Possible Missed Diagnoses of Subarachnoid Hemorrhage (SAH)

eTable 15. Manual Medical Record Review of Possible Missed Diagnoses of SAH With Secondary Testing

eTable 16. Possible Missed Diagnoses of Bacterial Meningitis

eTable 17. Manual Medical Record Review of Possible Missed Diagnoses of Bacterial Meningitis Following Secondary Testing

This supplemental material has been provided by the authors to give readers additional information about their work.

© 2024 Mark DG et al. *JAMA Network Open.*

eMethods

Study exclusion diagnostic codes

Diagnosis	ICD-9th revision codes	ICD-10th revision codes
Subarachnoid hemorrhage	430	I60.x
Unruptured cerebral aneurysm	437.3	I67.1
Arteriovenous malformation of the cerebral vessels	747.81	Q28.2
Cerebrospinal fluid shunt	V45.2	Z98.2

ICD = International Classification of Diseases

Diagnostic codes and hierarchical ranking of the primary emergency department diagnosis

Diagnosis	ICD-9th revision codes	ICD-10th revision codes	Rank
Subarachnoid hemorrhage	430	I60.x	1
Meningitis	3.21, 13.0x, 36, 47.0, 47.1, 47.8, 47.9, 49.1, 53, 54.72, 72.1, 90.42, 91.81, 94.2, 98.82, 100.81, 112.83, 114.2, 115.01, 115.11, 115.91, 320.x, 321.x, 322.x,	A01.01, A02.21, A17.0, A20.3, A27.81, A32.1, A32.11, A39.0, A42.81, A50.41, A51.41, A52.13, A54.81, A69.21, A87, A87.1, A87.8, A87.9, B00.3, B01.0, B02.1, B05.1, B06.02, B26.1, B27.02, B27.12, B27.82, B27.92, B37.5, B38.4, D86.81, G00.x, G01, G02, G03.x	2
Intracerebral hemorrhage	431	I61.x	3
Brain tumor	191.x, 225, 225.2, 784.2	C71.x, D32.0, D33.0, D33.1, D33.2, , R90.0	4
Ischemic stroke	434.x	I63.x	5
Hypertension	401, 401.1, 401.9	I10	6
Headache	339.x, 350.1,784	G44.x, G50.0, G50.1, G97.1, R51.0, R51.9	7
Upper respiratory infection	460-465, 466, 466.11, 466.19, 488.81, 488.82	J00-J06, J09.x, J20	8
Migrane	346.x	G43.x	9

ICD = International Classification of Diseases

Diagnostic codes for bacterial meningitis

ICD-9th revision codes	ICD-10th revision codes
3.21, 36, 98.82, 320, 320.1, 320.2, 320.3, 320.7, 320.8, 320.81, 320.82, 320.89, 320.9	A39.0, A54.81, G00, G00.0, G00.1, G00.2, G00.3, G00.8, G00.9, G01

ICD = International Classification of Diseases

Detection of unruptured intracranial aneurysms (UIA)

UIA detection was defined using two methods: 1) a problem list entry of UIA, using the date of entry as the diagnosis date and 2) natural language processing of the text of radiology reports potentially involving cranial

© 2024 Mark DG et al. *JAMA Network Open.*

vascular imaging (computed tomography, computed tomography cerebral angiography, magnetic resonance angiography or digital subtraction angiography) to identify suspected cerebral aneurysms. We used an iterative process and applied criterion to extracted findings and impressions text of radiology reports, with the goal of maximizing positive predictive value and arrived at the following method of UIA identification:

Using regular expression, we identified reports that included “aneur” (to cover aneurysm, aneurysmal, and potential shorthand) or “pouch”. We then isolated the specific sentences that included “aneur” or “pouch” and looked for a measurement within the same sentence. The combination of a measurement and “aneur” or “pouch” could be in either order; this allowed for the capture of variations of “a 5 mm aneurysm” or “an aneurysm measuring 5mm”. Measurements were defined in regular expression as a non-character string, followed by “mm” or “cm”, followed by a non-character. This allowed for capturing syntax such as: “5cm”,” 5 cm”, “4.2mm.”; but it would exclude words like “communicating”. Finally, we excluded sentences with “aort”, to avoid inclusion of aortic aneurysms.

This final methodology had a positive predictive value of 98.67% (97.58% to 99.28%) when applied to 1000 randomly selected radiology reports, as shown in the 2x2 table below.

	Presence of UIA	Absence of UIA
Positive natural language processing	743	10
Negative natural language processing	2	393

Sensitivity, n (%)	743/745 (99.7)
Specificity, n (%)	393/403 (97.5)
Positive predictive value, n (%)	743/753 (98.7)
Negative predictive value, n (%)	393/395 (99.5)

Determination of race/ethnicity categories

Race/ethnicity variables were extracted from existing electronic databases drawing from a pre-defined “race” designation within the electronic health record, in which a single entry is allowed for the following nine categories: American Indian/Alaska Native; Asian; Black/African American; Decline to state; Hispanic/Latino; Native Hawaiian/Other Pacific Islander; Other; Unknown; White.

eTable 1. Demographics, Baseline Characteristics, and Diagnoses: Emergency Department Encounters for Headache, 2015 to 2021, Full Study Cohort

© 2024 Mark DG et al. *JAMA Network Open.*

	No. (%)						
Year	2015	2016	2017	2018	2019	2020	2021
Encounters, n	27132	27944	28489	29179	31090	24942	29333
Age, mean (SD), y	46.6 (18.3)	46.6 (18.3)	47.2 (18.5)	47.6 (18.4)	47.8 (18.4)	48.5 (18.4)	48.3 (18.3)
Female	19605 (72.3)	20118 (72.0)	20337 (71.4)	20682 (70.9)	21779 (70.1)	17097 (68.6)	20383 (69.5)
Race/Ethnicity							
Asian	3138 (11.6)	3560 (12.7)	3766 (13.2)	3869 (13.3)	4367 (14.1)	3397 (13.6)	4145 (14.1)
Black/African American	4070 (15.0)	4461 (16.0)	4230 (14.9)	4363 (15.0)	4448 (14.3)	3412 (13.7)	4051 (13.8)
Hispanic/Latino	7518 (27.7)	7846 (28.1)	8299 (29.1)	8757 (30.0)	9753 (31.4)	7979 (32.0)	9744 (33.2)
White	11517 (42.5)	11148 (39.9)	11198 (39.3)	11115 (38.1)	11377 (36.6)	9107 (36.5)	10140 (34.6)
Other[a]	889 (3.3)	929 (3.3)	996 (3.5)	1075 (3.7)	1145 (3.7)	1047 (4.2)	1253 (4.3)
Body mass index, mean (SD), kg/m^2	28.7 (6.0)	28.7 (6.0)	28.8 (5.9)	29.0 (5.9)	29.1 (6.0)	29.1 (5.9)	29.3 (6.0)
Hypertension	10129 (37.3)	10198 (36.5)	10409 (36.5)	10959 (37.6)	11699 (37.6)	9746 (39.1)	11296 (38.5)
Smoking							
Active	1097 (4.0)	1449 (5.2)	1345 (4.7)	1298 (4.4)	1234 (4.0)	857 (3.4)	917 (3.1)
Former	3839 (14.2)	6053 (21.7)	6344 (22.3)	6422 (22.0)	6884 (22.1)	5533 (22.2)	6273 (21.4)
Never	17148 (63.2)	18050 (64.6)	18492 (64.9)	19201 (65.8)	20703 (66.6)	16445 (65.9)	19504 (66.5)
Unknown	5048 (18.6)	2392 (8.6)	2298 (8.1)	2258 (7.7)	2259 (7.3)	2107 (8.5)	2639 (9.0)
Diabetes	4446 (16.4)	4805 (17.2)	4987 (17.5)	5296 (18.2)	5849 (18.8)	5036 (20.2)	5475 (18.7)
Hyperlipidemia	9577 (35.3)	9589 (34.3)	9718 (34.1)	9882 (33.9)	10384 (33.4)	8602 (34.5)	9913 (33.8)

© 2024 Mark DG et al. *JAMA Network Open.*

Family history of CA	14 (0.1)	19 (0.1)	15 (0.1)	30 (0.1)	38 (0.1)	31 (0.1)	39 (0.1)
Temp > 100.4F	233 (0.9)	234 (0.8)	264 (0.9)	236 (0.8)	235 (0.8)	242 (1.0)	237 (0.8)
Systolic blood pressure, mean (SD), mmHg	129.4 (19.5)	129.7 (19.8)	130.2 (19.8)	130.7 (19.7)	130.8 (19.9)	132.5 (19.9)	133.1 (20.3)
Diastolic blood pressure, mean (SD), mmHg	74.1 (13)	74.1 (13.2)	74.5 (13.3)	74.8 (13.1)	74.8 (13.2)	75.8 (13.2)	76.6 (13.3)
Stroke alert activation in ED	4 (0.0)	97 (0.3)	126 (0.4)	190 (0.7)	213 (0.7)	191 (0.8)	209 (0.7)
Admission to hospital	1740 (6.4)	1807 (6.5)	1956 (6.9)	1962 (6.7)	2051 (6.6)	2024 (8.1)	2134 (7.3)
ED Diagnosis							
Headache	15151 (55.8)	16336 (58.5)	16639 (58.4)	17394 (59.6)	18685 (60.1)	15084 (60.5)	17518 (59.7)
Migrane	3746 (13.8)	3645 (13.0)	3171 (11.1)	3099 (10.6)	3163 (10.2)	2392 (9.6)	3131 (10.7)
SAH	96 (0.4)	125 (0.5)	137 (0.5)	144 (0.5)	138 (0.4)	140 (0.6)	121 (0.4)
Hypertension	1710 (6.3)	1402 (5.0)	1299 (4.6)	1237 (4.2)	1409 (4.5)	1286 (5.2)	1951 (6.7)
URI	775 (2.9)	730 (2.6)	802 (2.8)	811 (2.8)	856 (2.8)	615 (2.5)	610 (2.1)
Brain tumor	180 (0.7)	70 (0.3)	82 (0.3)	59 (0.2)	84 (0.3)	101 (0.4)	127 (0.4)
ICH	53 (0.2)	60 (0.2)	58 (0.2)	57 (0.2)	57 (0.2)	52 (0.2)	37 (0.1)
Meningitis	135 (0.5)	115 (0.4)	126 (0.4)	107 (0.4)	90 (0.3)	71 (0.3)	55 (0.2)
Ischemic stroke	462 (1.7)	311 (1.1)	459 (1.6)	700 (2.4)	782 (2.5)	602 (2.4)	173 (0.6)
Other	4824 (17.8)	5150 (18.4)	5716 (20.1)	5571 (19.1)	5826 (18.7)	4599 (18.4)	5610 (19.1)

[a] Includes American Indian/Alaska Native, Native Hawaiian/Other Pacific Islander, declined to state, unknown and “other”.
Abbreviations: CA = cerebral aneurysm; ED = emergency department; ICH = intracerebral hemorrhage; SAH = nontraumatic subarachnoid hemorrhage; URI = upper respiratory infection.

© 2024 Mark DG et al. *JAMA Network Open.*

eTable 2. Sensitivity Analysis: Trends in Test Utilization During Emergency Department Encounters for Headache, Excluding Stroke Alert Activations and 2015 Encounters

	**Year**	**2016**	**2017**	**2018**	**2019**	**2020**	**2021**	
Full study cohort	Encounters, n	27847	28363	28989	30877	24751	29124	
		Utilization rates (per 1000 encounters)						AAPC (95% CI)
	CT	317.7	342.4	366.3	381.0	389.3	412.3	+5.0 (+3.3 to +6.7)
	CTCA	23.8	31.5	36.2	45.1	51.6	55.2	+17.3 (+13.3 to +22.3)
	LP	34.2	31.2	29.1	25.5	21.8	18.6	-11.0 (-14.9 to -7.4)
	Either LP or CTCA	54.9	59.1	61.2	66.8	69.1	69.8	+5.1 (+3.1 to +7.0)
	Both LP and CTCA	3.1	3.6	4.1	3.8	4.3	4.0	+4.8 (-2.9 to +13.2)
	CTCA:LP ratio	0.7	1.0	1.2	1.8	2.4	3.0	+33.7 (+28.8 to +39.2)
	DSA (72 hours)	3.3	3.0	1.7	2.1	3.0	2.5	-4.7 (-16.6 to +6.2)
Diagnostic testing subcohort	**Year**	**2016**	**2017**	**2018**	**2019**	**2020**	**2021**	
	Encounters, n	9039	9920	10790	11907	9716	12079	
		Utilization rates (per 1000 encounters)						AAPC (95% CI)
	CT	978.8	978.9	984.1	987.9	991.7	994.0	+0.3 (+0.3 to +0.4)
	CTCA	73.5	90.0	97.2	116.9	131.4	133.0	+12.1 (+6.0 to +19.7)
	LP	105.2	89.1	78.1	66.2	55.6	44.9	-15.1 (-16.9 to -13.7)
	Either LP or CTCA	169.3	168.9	164.3	173.3	176.0	168.3	+0.4 (-1.7 to +2.3)
	Both LP and CTCA	9.4	10.3	11.0	9.8	11.0	9.6	-0.1 (-7.2 to +7.7)
	CTCA:LP ratio	0.7	1.0	1.2	1.8	2.4	3.0	+33.7 (+28.8 to +39.2)
	DSA (72 hours)	9.5	8.1	4.5	5.0	7.4	5.9	-8.5 (-20.4 to +2.6)

Abbreviations: AAPC = average annual percentage change; CT = non-contrast head computed tomography; CTCA = computed tomography cerebral angiography; DSA = digital subtraction invasive cerebral angiography; LP = lumbar puncture.

© 2024 Mark DG et al. *JAMA Network Open.*

eTable 3. Sensitivity Analysis: Comparison of Trends in Test Utilization During Emergency Department Encounters for Headache Using Joinpoint Analysis vs Poisson Regression Adjusted for Emergency Department-Level Effects

Cohort	Test	Average annual percentage change (joinpoint)	Annual percentage change (Poisson regression)*
Full cohort	CT	+5.4 (+5.1 to +5.8)	+5.4 (+5.1 to +5.7)
	CTCA	+18.8 (+17.7 to +20.3)	+18.4 (+17.2 to +19.7)
	LP	-11.1 (-12.0 to -10.4)	-10.7 (-11.9 to -9.5)
	Either LP/CTCA	+5.3 (+3.7 to +7.0)	+5.3 (+4.4 to +6.1)
	Both LP/CTCA	+6.4 (+3.6 to +10.1)	+5.9 (+2.2 to +9.7)
	DSA (72 hours)	-2.4 (-13.7 to +8.9)	-1.0 (-5.3 to +3.4)
Diagnostic testing subcohort	CT	+0.3 (+0.3 to +0.4)	+0.4 (+0.4 to +0.5)
	CTCA	+13.2 (+11.7 to + 15.8)	+13.0 (+11.8 to + 14.2)
	LP	-15.3 (-16.5 to -14.3)	-15.0 (-16.1 to -13.9)
	Either LP/CTCA	+0.4 (-1.5 to +2.4)	+0.3 (-0.5 to +1.1)
	Both LP/CTCA	+1.2 (-1.4 to +4.9)	+1.0 (-2.6 to +4.7)
	DSA (72 hours)	-6.1 (-17.9 to +6.2)	-4.9 (-9.2 to -0.5)

*Adjusted for between-facility (emergency department) differences. Abbreviations: CT = non-contrast head computed tomography; CTCA = computed tomography cerebral angiography; DSA = digital subtraction invasive cerebral angiography; GEE = general estimating equations; LP = lumbar puncture.

© 2024 Mark DG et al. *JAMA Network Open.*

eTable 4. Time Trends in Noncontrast Computed Tomography (CT) of the Head During Emergency Department Encounters for Headache (2015-2021), Stratified by Age

Year	2015	2016	2017	2018	2019	2020	2021	
	CT utilization rates (per 1000 encounters)							AAPC (95% CI)
Age 18-40	183.6	198.6	217.7	238.4	250.2	265.5	273.7	+6.9 (+6.6 to +7.3)
Age 41-60	300.6	320.4	337.1	371.6	387.7	379.3	417.0	+5.3 (+4.0 to +6.8)
Age 61-80	478.0	488.5	517.8	531.4	553.4	551.4	577.4	+3.1 (+1.9 to +4.3)
Age > 80	622.7	634.9	676.8	700.4	687.9	717.4	735.4	+2.8 (+2.3 to +3.5)

© 2024 Mark DG et al. *JAMA Network Open.*

eFigure 1. Time Trends in Noncontrast Computed Tomography (CT) of the Head, Stratified by Age

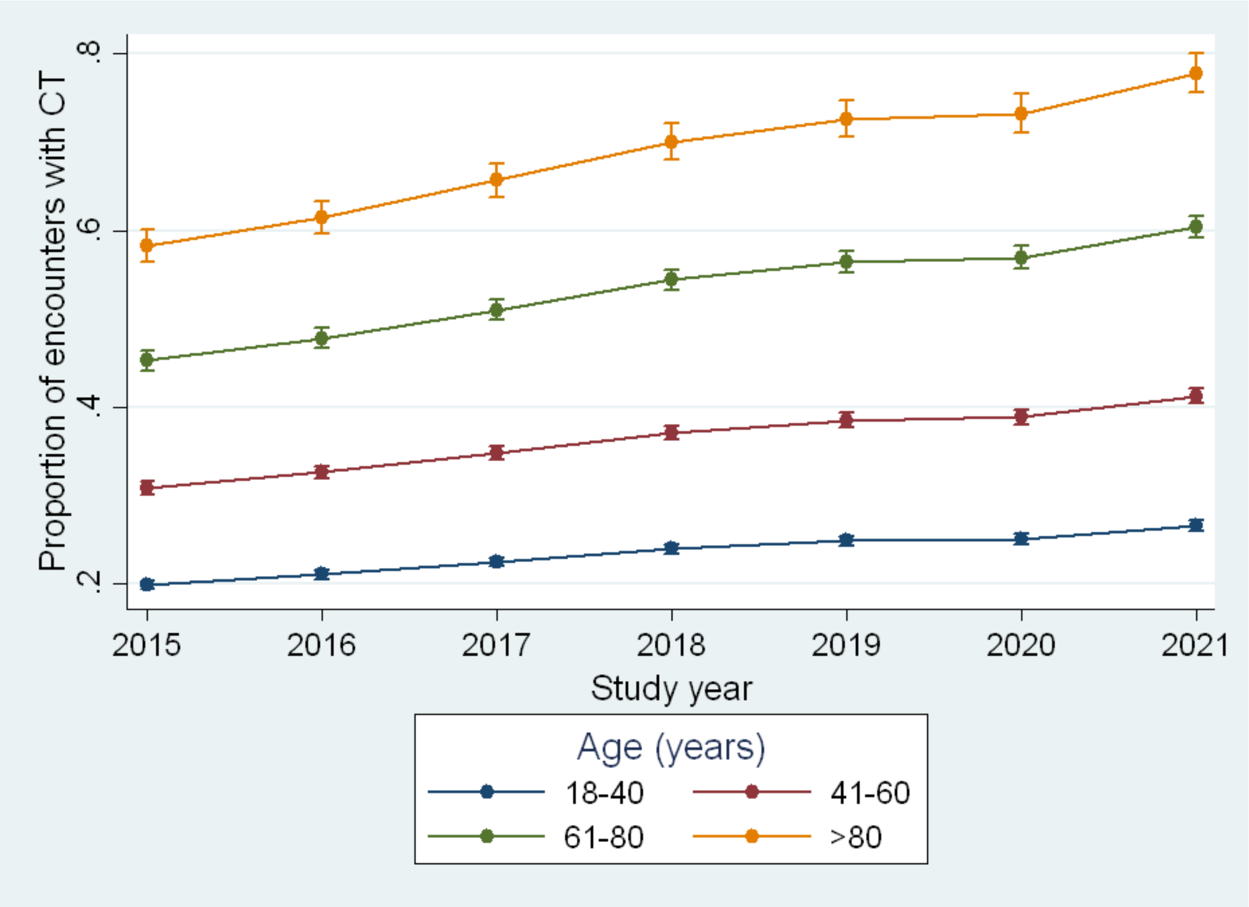

© 2024 Mark DG et al. *JAMA Network Open.*

eTable 5. Time Trends in Computed Tomography Cerebral Angiography (CTCA) During Emergency Department Encounters for Headache (2015-2021), Stratified by Age

**Year**	**2015**	**2016**	**2017**	**2018**	**2019**	**2020**	**2021**	
	CTCA utilization rates (per 1000 encounters)							AAPC (95% CI)
Age 18-40	10.3	12.7	17.0	21.6	26.6	31.6	32.1	+21.4<br>(+18.9 to +25.9)
Age 41-60	23.3	29.2	39.5	42.7	56.7	63.5	68.0	+19.0<br>(+17.4 to +21.9)
Age 61-80	39.2	46.5	58.4	66.0	75.8	81.4	85.6	+14.1<br>(+12.5 to +16.6)
Age > 80	23.3	35.2	45.4	57.8	63.1	75.0	84.7	+21.8<br>(+20.0 to +25.0)

© 2024 Mark DG et al. *JAMA Network Open.*

eFigure 2. Time Trends in Computed Tomography Cerebral Angiography (CTCA), Stratified by Age

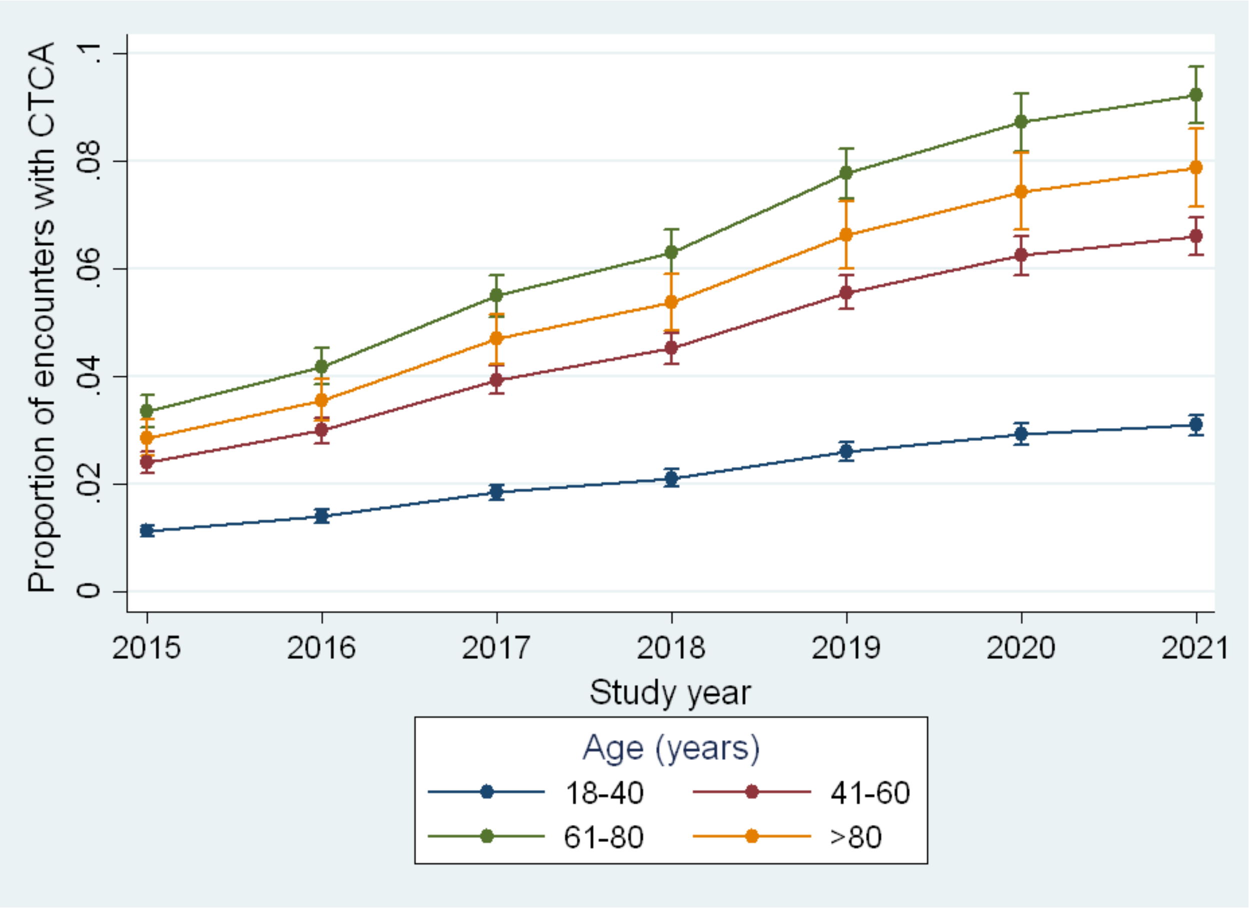

© 2024 Mark DG et al. *JAMA Network Open.*

eTable 6. Time Trends in Lumbar Puncture (LP) During Emergency Department Encounters for Headache (2015-2021), Stratified by Age

**Year**	**2015**	**2016**	**2017**	**2018**	**2019**	**2020**	**2021**	
	LP utilization rates (per 1000 encounters)							AAPC (95% CI)
Age 18-40	42.2	37.5	35.2	31.2	26.6	22.0	20.3	-11.9<br>(-14.1 to -10.3)
Age 41-60	39.8	39.6	33.2	32.5	30.0	23.9	20.2	-11.2<br>(-13.8 to -9.0)
Age 61-80	32.7	21.5	24.1	23.2	19.9	19.9	15.7	-9.4<br>(-13.9 to -5.0)
Age > 80	16.0	12.2	11.8	13.1	8.9	13.5	10.3	-3.5<br>(-15.8 to +10.2)

© 2024 Mark DG et al. *JAMA Network Open.*

eFigure 3. Time Trends in Lumbar Puncture (LP), Stratified by Age

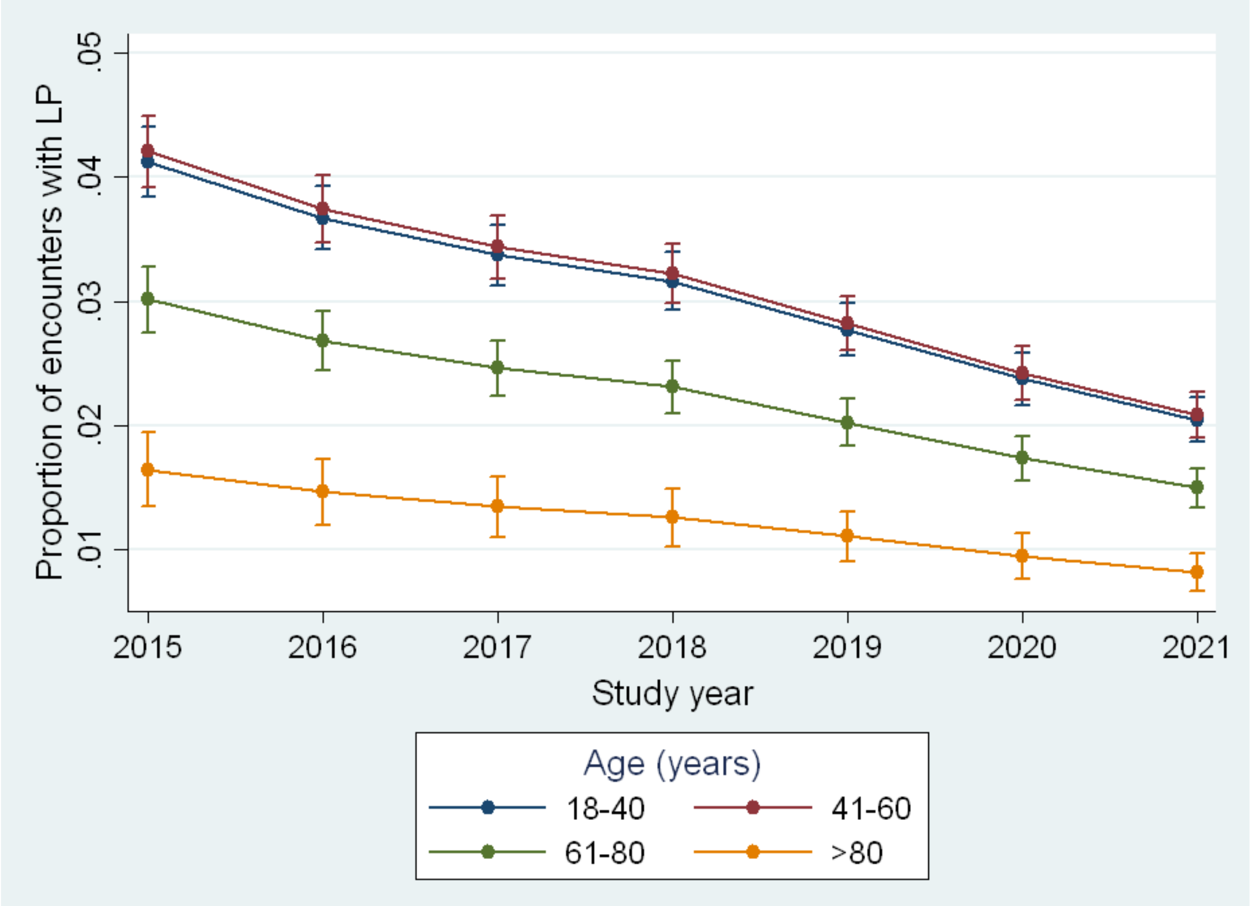

© 2024 Mark DG et al. *JAMA Network Open.*

eFigure 4. Days From Index ED Encounter to Subarachnoid Hemorrhage (SAH) Diagnosis

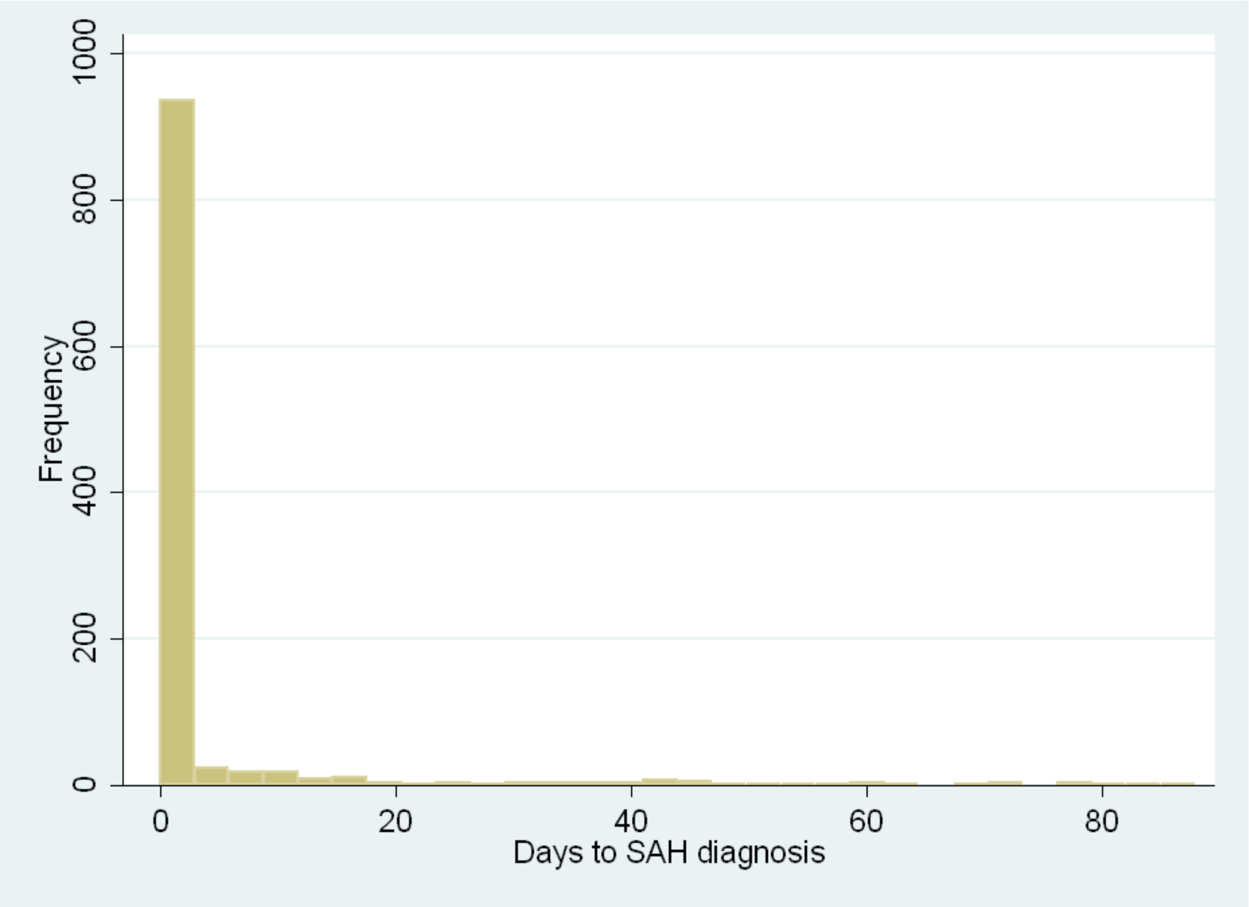

© 2024 Mark DG et al. *JAMA Network Open.*

eFigure 5. Days From Index ED Encounter to Unruptured Intracranial Aneurysm (UIA) Diagnoses

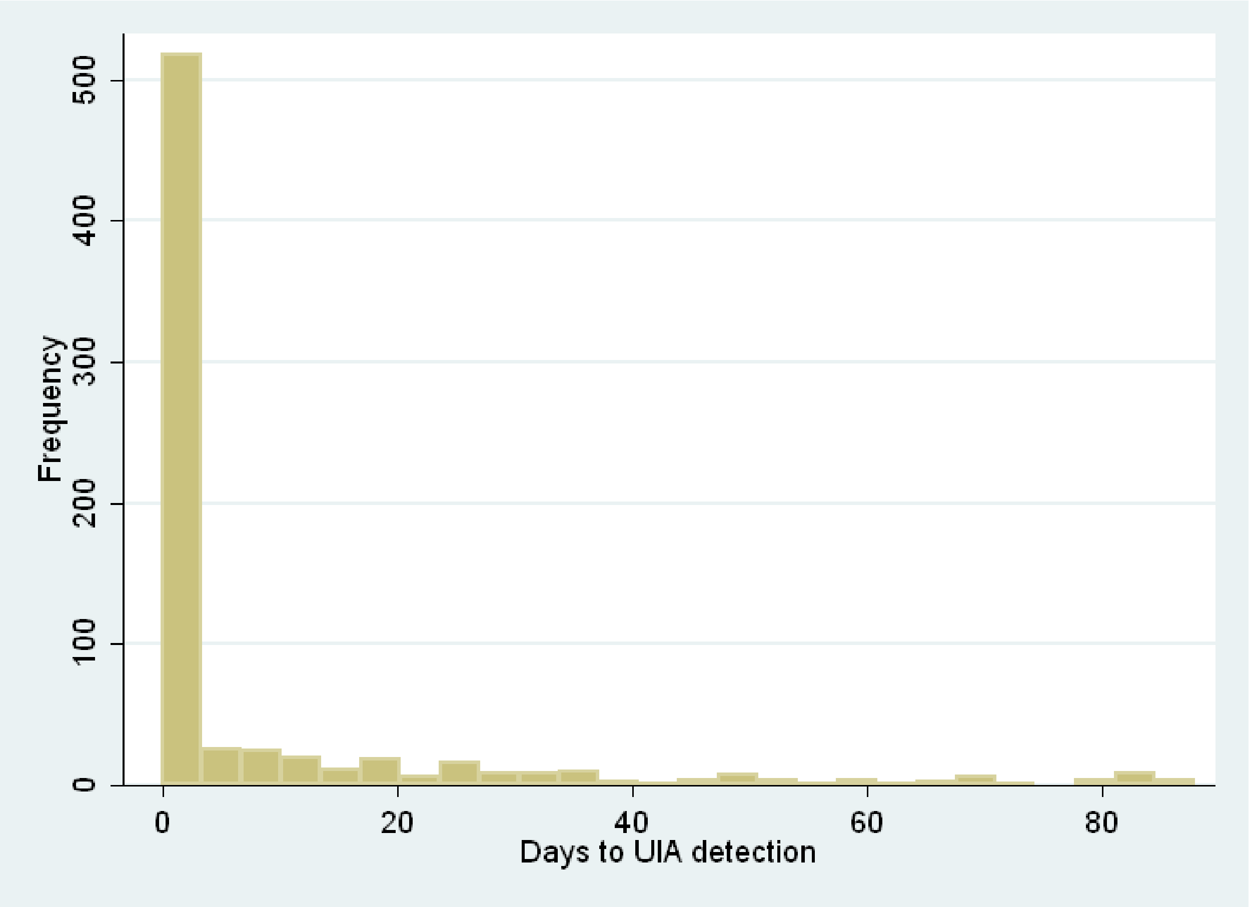

© 2024 Mark DG et al. *JAMA Network Open.*

eTable 7. Sensitivity Analysis: Primary and Secondary Outcomes, Excluding Stroke Alert Activations and 2015 Encounters

	Year	2016	2017	2018	2019	2020	2021	
Full study cohort	Persons, n	24124	25000	25609	27365	22300	26115	
		14-day incidence (per 1000 persons)						AAPC (95% CI)
	UIA	2.7	2.5	3.4	2.9	4.1	3.9	+9.0% (-1.7 to +23.4%)
	SAH	5.7	5.8	6.1	5.2	6.5	5.1	-0.4% (-8.7 to +8.1%)
	UIA:SAH ratio	0.5	0.4	0.6	0.6	0.6	0.8	+10.7 (+2.4 to +20.9)
		90-day incidence (per 1000 persons)						AAPC (95% CI)
	UIA	3.5	3.2	3.8	3.7	4.8	4.6	+7.3% (+0.8 to 15.7%)
	SAH	6.1	6.2	6.4	5.6	6.9	5.4	-0.8% (-9.1 to +8.3%
	UIA:SAH ratio	0.6	0.5	0.6	0.7	0.7	0.9	+15.2 (+8.0 to +24.1)
Diagnostic testing subcohort	Year	2016	2017	2018	2019	2020	2021	
	Persons, n	8245	9099	9899	10884	8937	11118	
		14-day incidence (per 1000 persons)						AAPC (95% CI)
	UIA	5.6	5.9	7.4	6.4	8.8	8.6	+9.3% (+1.6 to +20.0%)
	SAH	15.3	15.5	14.4	12.2	15.2	11.5	-4.1% (-11.3 to +3.6%)
	UIA:SAH ratio	0.4	0.4	0.5	0.5	0.6	0.8	+9.3 (+4.4 to +14.6)
		90-day incidence (per 1000 persons)						AAPC (95% CI)
	UIA	6.8	7.3	8.1	7.7	10.2	9.7	+8.2% (+4.4 to +13.0%)
	SAH	16.4	16.4	15.3	13.0	15.8	12.1	-4.4% (-10.8 to +2.9%)
	UIA:SAH ratio	0.4	0.4	0.5	0.6	0.6	0.8	+14.0 (+10.9 to +17.7)

Abbreviations: AAPC = averaged annual percentage change; CT = non-contrast head computed tomography; SAH = non-traumatic subarachnoid hemorrhage; UIA = unruptured intracranial aneurysm.

© 2024 Mark DG et al. *JAMA Network Open.*

eTable 8. Sensitivity Analysis: Unruptured Intracranial Aneurysm (UIA) Detection by Problem List Only

Year	2015	2016	2017	2018	2019	2020	2021	
Persons, n	23010	24215	25118	25785	27554	22484	26312	
	14-day incidence (per 1000 persons)							AAPC (95% CI)
Diagnosis by problem list	1.3	1.3	1.2	1.5	1.4	1.6	2.1	+8.4% (+5.8 to +11.3%)
Revised UIA:SAH ratio	0.3	0.2	0.2	0.2	0.3	0.2	0.4	+4.3% (0.0 to +12.5%)
	90-day incidence (per 1000 persons)							
Diagnosis by problem list	1.3	1.7	1.6	1.7	1.6	2.0	2.5	+8.8% (+4.7 to +13.5%)
Revised UIA:SAH ratio	0.3	0.3	0.3	0.3	0.3	0.3	0.4	+7.4% (+2.3 to +13.5%)

Rates of unruptured intracranial aneurysms were restricted to diagnoses that populated the problem list. Revised UIA:SAH ratios using the problem list verified UIA rates are provided.

Abbreviations: AAPC = average annual percentage change; SAH = subarachnoid hemorrhage; UIA = unruptured intracranial aneurysm.

© 2024 Mark DG et al. *JAMA Network Open.*

eTable 9. Sensitivity Analysis: Risk-Adjusted Unruptured Intracranial Aneurysm (UIA) Rates Among Emergency Department Patients With Headache, 2015 to 2021

Year	2015	2016	2017	2018	2019	2020	2021	
	Adjusted incidence per 1000 persons							APC (95% CI)
14-day UIA, full study cohort	3.2	3.0	2.7	3.5	3.0	4.3	4.0	+5.8 (+1.4 to +10.3)
90-day UIA, full study cohort	3.7	3.9	3.4	3.9	3.9	5.0	4.6	+5.0 (+1.1 to +9.1)
14-day UIA, diagnostic testing subcohort	6.7	6.2	6.4	7.6	6.8	9.4	8.9	+6.7 (+2.0 to +11.8)
90-day UIA, diagnostic testing subcohort	7.0	7.6	7.7	8.3	8.2	10.7	9.9	+6.6 (+2.2 to +11.2)

Rates of unruptured intracranial aneurysms were risk-adjusted using marginal standardization with Poisson regression and covariates of age, sex, race/ethnicity, diabetes, hypertension, smoking status, and family history of cerebral aneurysms. The models also included dummy variables for each emergency department to account for any bias from between-ED differences.

Abbreviations: APC = annual percentage change; SAH = non-traumatic subarachnoid hemorrhage; UIA = unruptured intracranial aneurysm.

© 2024 Mark DG et al. *JAMA Network Open.*

eTable 10. Sensitivity Analysis: Comparison of Trends in Test Utilization During Emergency Department Encounters for Headache Using Joinpoint Analysis Versus Poisson Regression Adjusted for Emergency Department-Level Effects

Cohort	Outcome	Average annual percentage change (joinpoint)	Annual percentage change (Poisson regression*)
Full cohort	14d UIA	+7.5% (+1.2% to +16.2%)	+7.2% (+2.8% to +11.8%)
	14d SAH	+2.4% (-5.6% to +12.0%)	+2.5% (-0.6% to +5.7%)
	90d UIA	+6.6% (+1.2% to +13.6%)	+6.4% (+2.4% to +10.4%)
	90d SAH	+2.0% (-5.0% to +10.6%)	+2.3% (-0.7% to +5.3%)
Diagnostic testing subcohort	14d UIA	+7.4% (+2.2% to +14.9%)	+7.3% (+2.5% to +12.4%)
	14d SAH	-1.3% (-10.2% to +10.4%)	-1.6% (-4.7% to +1.5%)
	90d UIA	+7.1% (+4.2% to +10.9%)	+7.1% (+2.7% to +11.7%)
	90d SAH	-1.6% (-9.4% to +9.1%)	-1.7% (-4.7% to +1.3%)

*Adjusted for between-facility (emergency department) differences. Abbreviations: SAH = non-traumatic subarachnoid hemorrhage; UIA = unruptured intracranial aneurysm.

© 2024 Mark DG et al. *JAMA Network Open.*

eTable 11. Average Percentage Change (APC) in Test Utilization by Joinpoint Segment, Primary Analysis

Cohort	Test	Seg 1 time	Seg 1 APC	Seg 2 time	Seg 2 APC
Full study cohort	CT	2015-2018	+7.0 (+6.2 to +8.2)	2018-2021	+3.9 (+3.0 to +4.6)
	CTCA	2015-2019	+24.2 (+21.9 to +29.0)	2019-2021	+8.6 (+5.5 to +13.2)
	LP	2015-2019	-9.32 (-10.2 to -7.18)	2019-2021	-14.7 (-17.6 to -12.0)
	Either CTCA or LP	2015-2021	+5.3 (+3.7 to +7.0)	NA	NA
	Both CTA and LP	2015-2018	+13.1 (+7.7 to +28.2)	2018-2021	+0.1 (-8.2 to +4.8)
	CTCA:LP ratio	2015-2017	+42.2 (+34.9 to +50.7)	2017-2021	+31.5 (+25.3 to +34.5)
	DSA	2015-2021	-2.4 (-13.7 to +8.9)	NA	NA
Diagnostic testing subcohort	CT	2015-2021	+0.3 (+0.3 to +0.4)	NA	NA
	CTCA	2015-2019	+17.3 (+14.5 to +25.4)	2019-2021	+13.2 (+11.7 to +15.8)
	LP	2015-2019	-14.4 (-16.5 to -11.0)	2019-2021	-17.1 (-20.8 to -14.1)
	Either CTCA or LP	2015-2021	+0.4 (-1.5 to +2.4)	NA	NA
	Both CTA and LP	2015-2018	+6.0 (-1.2 to +20.4)	2018-2021	-3.4 (-11.3 to +2.7)
	CTCA:LP ratio	2015-2017	+42.2 (+34.9 to +50.7)	2017-2021	+31.5 (+25.3 to +34.5)
	DSA	2015-2021	-6.1 (-17.9 to +6.2)	NA	NA

Abbreviations: APC = average percentage change; CT = non-contrast head computed tomography; CTCA = computed tomography cerebral angiography; DSA = digital subtraction invasive cerebral angiography; LP = lumbar puncture.

© 2024 Mark DG et al. *JAMA Network Open.*

eTable 12. Average Percentage Change (APC) in Outcomes by Joinpoint Segment, Primary Analysis

Full study cohort	UIA (14d)	2015-2021	+7.5 (+1.2 to +16.2)	NA	NA
	SAH (14d)	2015-2021	+2.4 (-5.6 to +12.0)	NA	NA
	UIA:SAH ratio (14d)	2015-2017	-11.9 (-19.3 to +0.5)	2017-2021	+12.1 (+8.4 to +22.5)
	UIA (90d)	2015-2021	+6.6 (+1.2 to +13.6	NA	NA
	SAH (90d)	2015-2021	+2.0 (-5.0 to +10.6)	NA	NA
	UIA:SAH ratio (90d)	2015-2017	-11.5 (-13.5 to -9.0)	2018-2021	+11.1 (+9.9 to +12.2)
Diagnostic subset	UIA (14d)	2015-2021	+7.4 (+2.2 to +14.9)	NA	NA
	SAH (14d)	2015-2021	-1.3 (-10.2 to +10.4)	NA	NA
	UIA:SAH ratio (14d)	2015-2017	-4.9 (-16.7 to +20.8)	2017-2021	+14.8 (+3.2 to +34.1)
	UIA (90d)	2015-2021	+7.1 (+4.2 to +10.9)	NA	NA
	SAH (90d)	2015-2021	-1.6 (-9.4 to +9.1)	NA	NA
	UIA:SAH ratio (90d)	2015-2017	-2.7 (-8.4 to +10.1)	2017-2021	+13.7 (+10.2 to +23.4)

Abbreviations: APC = average percentage change; CT = non-contrast head computed tomography; CTCA = computed tomography cerebral angiography; DSA = digital subtraction invasive cerebral angiography; LP = lumbar puncture.

© 2024 Mark DG et al. *JAMA Network Open.*

eFigure 6. Correlation Between Annualized CTCA:LP Ratios and 14-Day UIA:SAH Ratios (Full Study Cohort)

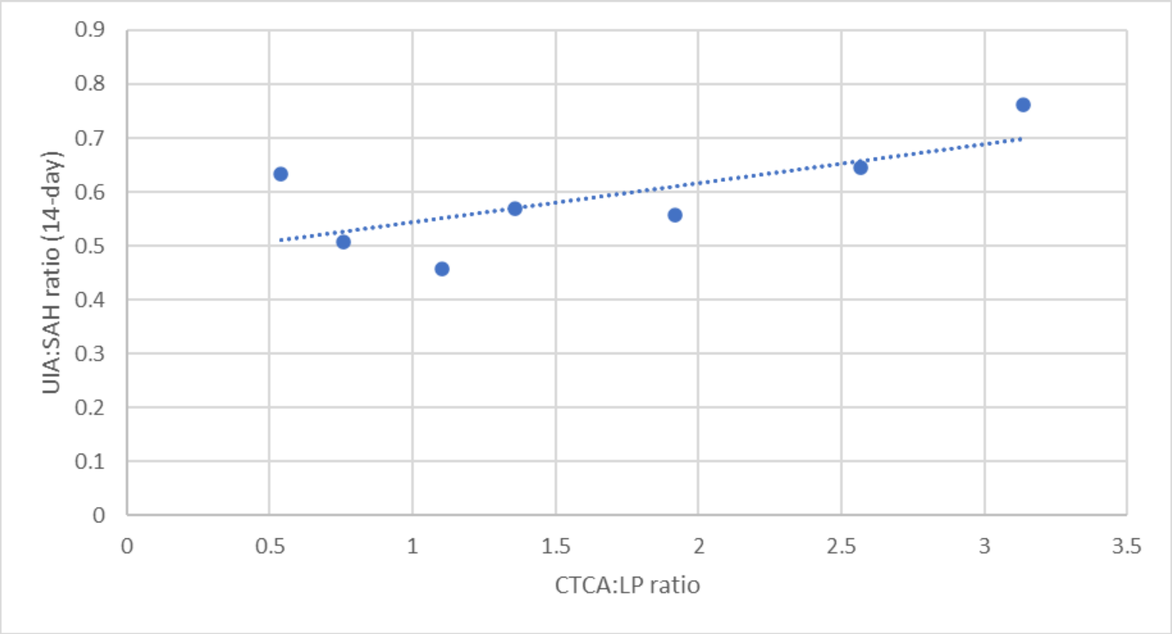

© 2024 Mark DG et al. *JAMA Network Open.*

eFigure 7. Correlation Between Annualized CTCA:LP Ratios and 14-Day UIA:SAH Ratios (Diagnostic Testing Subcohort)

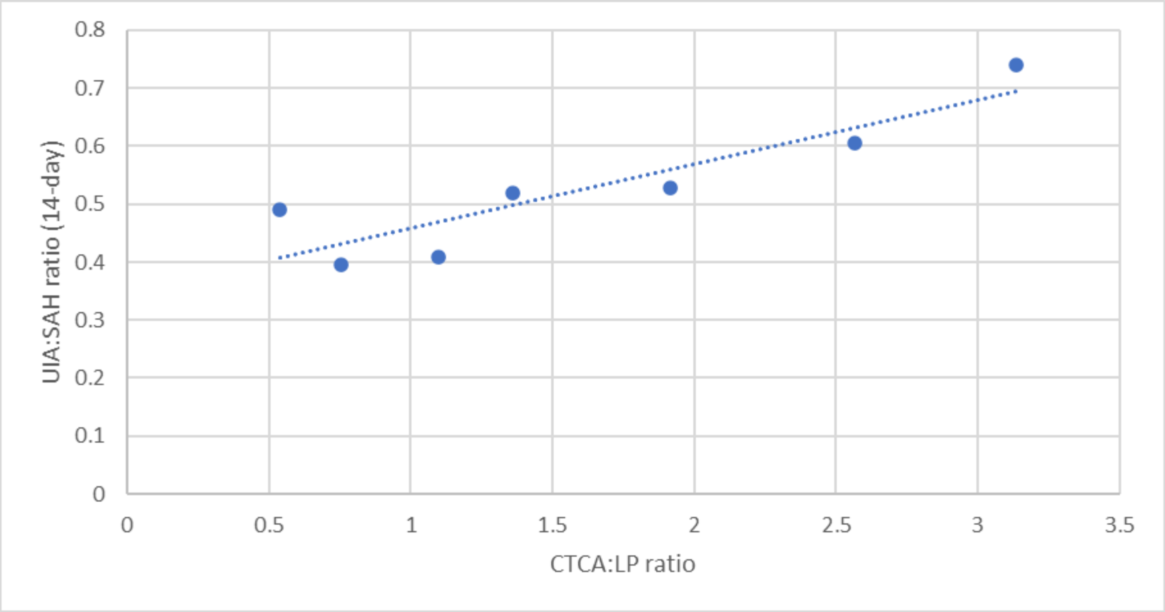

Pearson's correlation coefficients (r) = 0.70 (Figure 2a) and 0.90 (Figure 2b).

Abbreviations: CTCA = computed tomographic cerebral angiography; LP = lumbar puncture; SAH = subarachnoid hemorrhage; UIA = unruptured intracranial aneurysm.

eFigure 8. Correlation Between Annualized CTCA:LP Ratios and 14-Day UIA:SAH Ratios (Full Study Cohort, Sensitivity Analysis Excluding Stroke Alert Activations and 2015 Encounters)

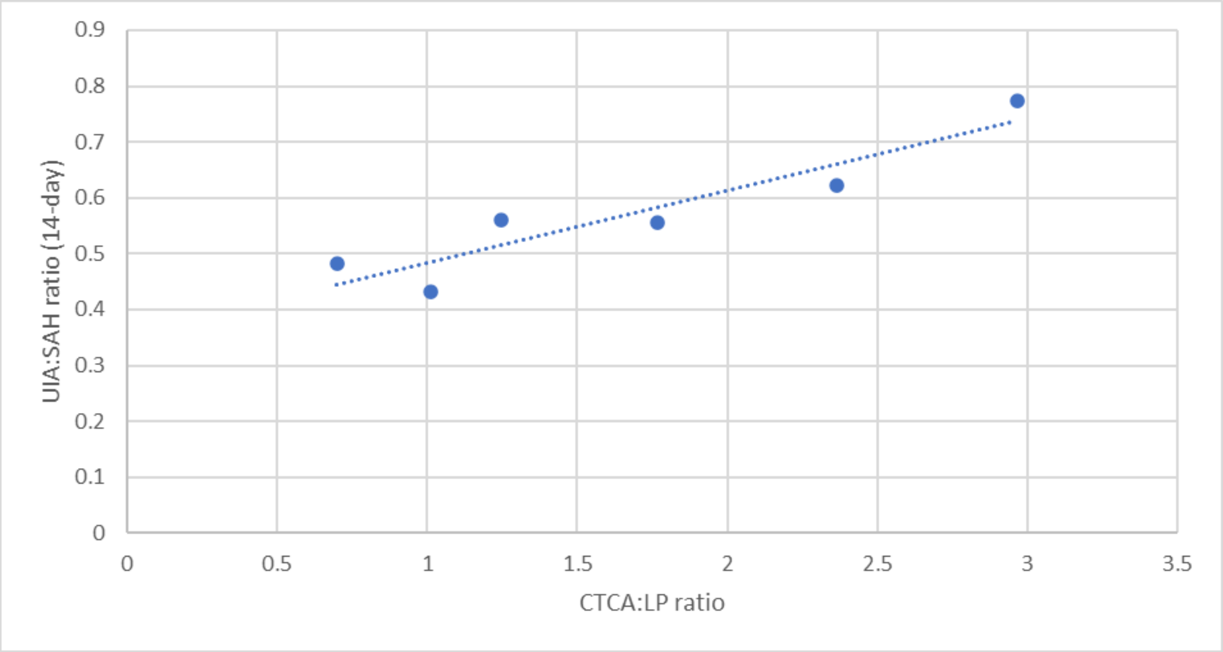

© 2024 Mark DG et al. *JAMA Network Open.*

eFigure 9. Correlation Between Annualized CTCA:LP Ratios and 14-Day UIA:SAH Ratios (Diagnostic Testing Subcohort, Sensitivity Analysis Excluding Stroke Alert Activations and 2015 Encounters)

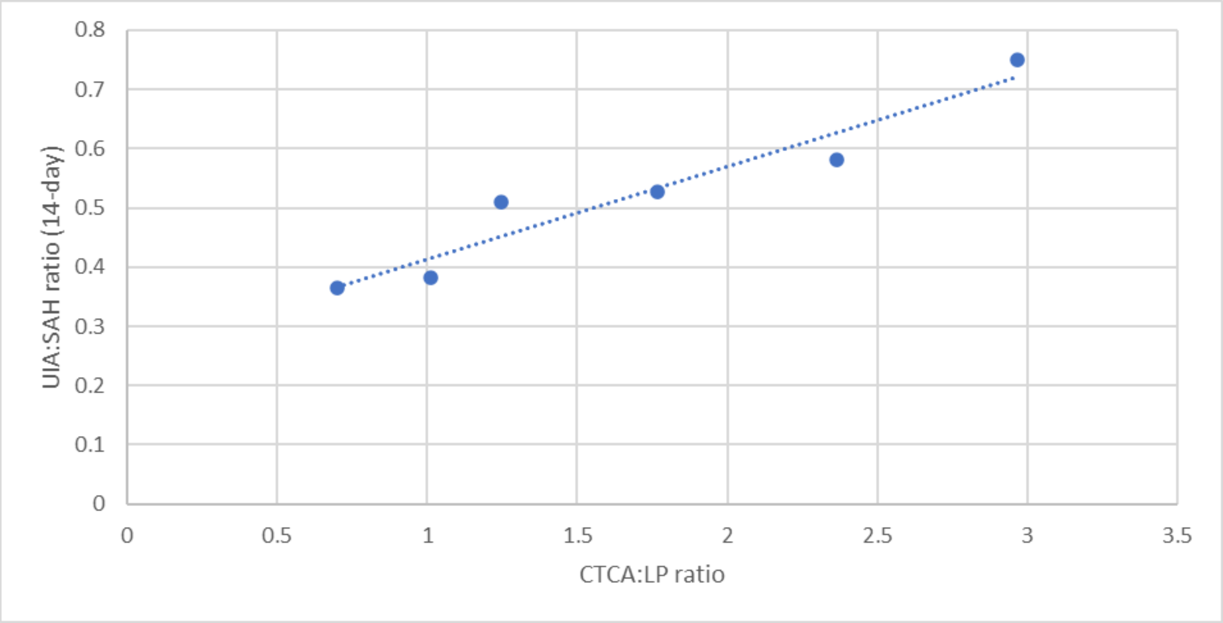

Pearson's correlation coefficients (r) = 0.93 (Figure e8) and 0.96 (Figure e9).

Abbreviations: CTCA = computed tomography cerebral angiography; LP = lumbar puncture; SAH = subarachnoid hemorrhage; UIA = unruptured intracranial aneurysm.

© 2024 Mark DG et al. *JAMA Network Open.*

eTable 13. Diagnostic Testing During ED Encounters With Study Outcomes Within 1 Calendar Day

	No. (%)						
Year	2015	2016	2017	2018	2019	2020	2021
SAH, n	96	125	137	144	138	140	121
CT at index visit	90 (94)	118 (94.4)	132 (96.4)	138 (95.8)	133 (96.4)	134 (95.7)	119 (98.3)
CTCA at index visit *	44 (46)	63 (50.4)	75 (54.7)	73 (50.7)	86 (62.3)	98 (70.0)	89 (73.6)
LP at index visit	10 (10)	11 (8.8)	12 (8.8)	14 (9.7)	13 (9.4)	10 (7.1)	6 (5.0)
UIA detection, n	52	59	55	74	78	88	91
CT at index visit, n (%)	39 (75)	43 (73)	49 (89)	69 (93)	71 (91)	79 (90)	88 (97)
CTCA at index visit, n (%) *	23 (44)	24 (41)	32 (58)	47 (64)	61 (78)	71 (81)	75 (82)
LP at index visit, n (%)	11 (21)	9 (15)	10 (18)	13 (18)	11 (14)	22 (25)	12 (13)

Abbreviations: CT = non-contrast head computed tomography; CTCA = computed tomography cerebral angiography; LP = lumbar puncture; SAH = subarachnoid hemorrhage; UIA = unruptured intracranial aneurysm.
* p for test of trend <0.001

© 2024 Mark DG et al. *JAMA Network Open.*

eTable 14. Possible Missed Diagnoses of Subarachnoid Hemorrhage (SAH)

	No. (%)							
Year	2015	2016	2017	2018	2019	2020	2021	Total
SAH, n	109	140	149	160	151	152	143	1004
ED visit for headache within 14 days prior to SAH with discharge to home	8 (7.3)	7 (5.0)	5 (3.4)	10 (6.3)	7 (4.6)	5 (3.3)	8 (5.6)	50 (5)*
No testing for SAH	1 (0.9)	3 (2.1)	1 (0.7)	4 (2.5)	2 (1.3)	2 (1.3)	1 (0.7)	14/50 (28)
CT only at index ED visit, n	6 (5.5)	2 (1.4)	3 (2.0)	5 (3.1)	5 (3.3)	1 (0.7)	7 (4.9)	29/50 (58)
CTCA at index ED visit, n	1 (0.9)	2 (1.4)	1 (0.7)	1 (0.6)	0 (0.0)	1 (0.7)	0 (0.0)	6/50 (12)
LP at index ED visit, n	0 (0.0)	0 (0.0)	0 (0.0)	0 (0.0)	0 (0.0)	1 (0.7)	0 (0.0)	1/50 (2)

Abbreviations: CT = non-contrast head computed tomography; CTCA = computed tomography cerebral angiography; LP = lumbar puncture; SAH = subarachnoid hemorrhage.

* P for test of trend = 0.34

© 2024 Mark DG et al. *JAMA Network Open.*

eTable 15. Manual Medical Record Review of Possible Missed Diagnoses of SAH With Secondary Testing

**Possible missed diagnosis index encounters with CTCA (6):**<br>- CT negative for SAH but CTCA positive for aneurysm but patient refused LP, subsequently returned with CT-positive SAH and underwent endovascular coiling (2)*<br>- CT and CTCA read as negative for SAH or cerebral aneurysm, subsequent return for CT-positive SAH with confirmed aneurysm rupture (1)<br>- Possible mass or basilar artery aneurysm on CT, no mass or vascular abnormality seen on CTCA or MRI/MRA, thought perhaps to represent atypical perimesencephalic SAH in retrospect (1)<br>- Subacute subdural hemorrhage which was later coded as SAH on a follow-up outpatient visit (1)<br>- Negative CT-CTA on initial evaluation, follow-up outpatient MRI/MRA noted focal hemosiderin deposits possibly representative of prior hemorrhage, no vascular abnormalities noted (1)
**Possible missed diagnosis index encounter with LP (1):**<br>- Negative CT and LP (for SAH) at index encounter, underwent subsequent neurosurgery for meningioma with SAH noted by CT on postoperative follow-up (1)

Abbreviations: CT = non-contrast head computed tomography; CTCA = computed tomography cerebral angiography; LP = lumbar puncture; MRI = magnetic resonance imaging; MRA = magnetic resonance angiography; SAH = subarachnoid hemorrhage.

eTable 16. Possible Missed Diagnoses of Bacterial Meningitis

	No. (%)							
Year	2015	2016	2017	2018	2019	2020	2021	Total
Bacterial meningitis, n	12	18	26	27	16	12	7	118
ED visit for headache within 7 days prior to bacterial meningitis with discharge to home, n (%)	4 (33)	3 (17)	3 (12)	5 (19)	2 (13)	2 (17)	2 (29)	21 (18)*
No testing, n	2 (17)	1 (6)	0 (0.0)	3 (11)	2 (13)	1 (8)	1 (14)	10 (9)
CT only at index ED visit, n	2 (17)	1 (6)	3 (12)	2 (7)	0 (0.0)	1 (8)	1 (14)	9 (8)
CTCA at index ED visit, n	0 (0.0)	0 (0.0)	0 (0.0)	0 (0.0)	0 (0.0)	0 (0.0)	0 (0.0)	0 (0.0)
LP at index ED visit, n	0 (0.0)	1 (6)	0 (0.0)	0 (0.0)	0 (0.0)	0 (0.0)	0 (0.0)	1 (1)

Abbreviations: CT = non-contrast head computed tomography; CTCA = computed tomography cerebral angiography; LP = lumbar puncture.

* p for test of trend = 0.73

© 2024 Mark DG et al. *JAMA Network Open.*

eTable 17. Manual Medical Record Review of Possible Missed Diagnoses of Bacterial Meningitis Following Secondary Testing

**Possible missed diagnosis index encounter with LP (1):**<br>- Underwent LP, CSF had 1-2 wbc, normal protein, slightly elevated glucose and negative gram stain. Returned 12 hours later with obtundation, repeat LP gram stain showed gram-positive cocci in pairs. CSF culture from initial visit ultimately had light growth of streptococcus pneumoniae.

Abbreviations: CSF = cerebrospinal fluid; LP = lumbar puncture.

© 2024 Mark DG et al. *JAMA Network Open.*
