# Supplementary material for: Shifts in Diagnostic Testing for Headache in the Emergency Department, 2015 to 2021
Source: JAMA Netw Open. 2024 Apr 19;7(4):e247373. doi: 10.1001/jamanetworkopen.2024.7373 (PMC11031686; doi:10.1001/jamanetworkopen.2024.7373)
Supplement: Supplement 2. — Data Sharing Statement [file jamanetwopen-e247373-s002.pdf]

Data Sharing Statement

Mark. Shifts in Diagnostic Testing for Headache in the Emergency Department, 2015 to 2021. *JAMA Netw Open*. Published April 19, 2024. doi:10.1001/jamanetworkopen.2024.7373

Data
Data available: No
